# Multi-Sensor SLAM and 3D Robotic Mapping in Confined Environments: A Systematic Review of Perception, Localization, and Autonomous Exploration

**DOI:** 10.3390/s26217070

**Published:** 2026-11-09

**Authors:** Ting Li, Tang Sai Hong, Azizan bin As’arry, Chenyuhao Du

**Affiliations:** Faculty of Engineering, Universiti Putra Malaysia, Serdang 43400, Selangor, Malaysia; gs75127@student.upm.edu.my (T.L.); zizan@upm.edu.my (A.b.A.); gs74769@student.upm.edu.my (C.D.)

**Keywords:** localization and mapping (SLAM), multi-modal sensor fusion, autonomous exploration, semantic scene understanding, multi-robot coordination, bibliometric synthesis

## Abstract

**Highlights:**

**What are the main findings?**

- This article systematically synthesizes data from 166 studies on robotic mapping in confined spaces, utilizing the PRISMA framework.
- Bibliometric analysis identifies SLAM, LiDAR, and multi-sensor fusion as the predominant research themes in recent years.
- Following bibliometric analysis, multidimensional comparisons identified significant gaps in semantic understanding, risk assessment, and autonomous decision-making.

**What are the implications of the main findings?**

- A three-axis framework links scene complexity, autonomy capability, and deployment maturity.
- Future priorities include semantic mapping, risk-aware autonomy, and multi-robot collaboration.

**Abstract:**

Low illumination, structural repetition, absence of satellite-based positioning signals, and communication constraints in confined spaces pose significant challenges for robotic localization, 3D mapping, and autonomous exploration. Following PRISMA 2020 reporting guidelines, this review included 166 studies identified through searches of Web of Science, Scopus, IEEE Xplore, and ScienceDirect. Bibliometric analyses—including publication and citation statistics, keyword co-occurrence, cluster classification, and temporal evolution—were conducted using VOSviewer, CiteSpace, and Bibliometrix. These quantitative approaches were complemented by content analysis to evaluate application scenarios, technical capabilities, and validation maturity. The findings indicate that existing research predominantly focuses on Simultaneous Localization and Mapping (SLAM), LiDAR perception, and multi-sensor fusion, with a strong emphasis on geometric mapping and localization. Conversely, investigations into semantic understanding, autonomous decision-making, risk assessment, and safe return mechanisms remain relatively insufficient. Validation methodologies are primarily limited to simulations and laboratory tests, resulting in scarce empirical evidence regarding real-world scenarios and long-term deployments. Based on these insights, this paper discusses technological evolution through the lens of scenario complexity, autonomy, and deployment maturity, and identifies prospective research directions such as semantic mapping, embodied intelligence, foundation models, multi-robot collaboration, risk-aware navigation, and standardized testing platforms. This review provides a reference framework for advancing robots in enclosed environments from geometric perception toward autonomous exploration.

## 1. Introduction

Constrained spaces are commonly found in underground mines, transportation tunnels, subterranean pipelines, building interiors, natural caves, and post-disaster collapse zones. These environments typically feature narrow spaces, complex structures, poor lighting, severe interference from dust or smoke, absence of global navigation satellite signals, and unstable communication links [1,2]. Manual entry for surveying, inspection, and search missions is not only inefficient but also poses significant safety risks such as collapses, hazardous gases, and secondary disasters. With advances in LiDAR, digital photogrammetry, visual sensors, and 3D reconstruction technologies, mapping in constrained spaces has gradually shifted from traditional manual control point measurements and contour tracing to automated perception and 3D mapping using mobile robots, offering safer and more efficient data acquisition methods for mine inspection, underground infrastructure monitoring, cave surveying, and disaster rescue [3,4,5,6].

Simultaneous Localization and Mapping (SLAM) is a core technology enabling autonomous operation of robots in constrained spaces. Robots must estimate their own pose in real time and simultaneously construct a 3D map of the surrounding environment under conditions where external positioning information is unavailable [7,8,9]. However, common challenges in constrained spaces—such as repetitive structures, geometric degeneracy, dynamic occlusions, and limited sensor field-of-view—often lead to odometry drift, loop closure failure, and map inconsistencies [10,11,12]. To improve localization accuracy and environmental adaptability, current research has evolved from single-sensor approaches based on LiDAR or vision to multi-sensor fusion systems integrating LiDAR, cameras, inertial measurement units, and wheel encoders. Related studies have investigated LiDAR–inertial SLAM using point cloud structure and appearance [13], as well as dynamic weighted fusion of multiple sensor sources in complex underground spaces [14].

This evolution further extends into semantic mapping, autonomous exploration, and collaborative multi-robot mapping. As a result, a comprehensive technical framework is emerging, centered on SLAM algorithms, supported by multi-sensor fusion, represented through 3D point clouds, and advancing toward autonomous navigation and multi-robot collaboration.

Despite rapid growth in recent years, existing research primarily focuses on “how robots localize” and “how they build maps,” with relatively insufficient attention given to “how robots understand their environment, recognize risks, and act autonomously.” Most reviews concentrate on specific SLAM algorithms, particular sensor combinations, underground robot platforms, or certain application scenarios [15,16,17], lacking a comprehensive evaluation of application distribution, robotic autonomy levels, and maturity of real-world deployment. To address this gap, this paper follows the PRISMA methodology, employing a combination of bibliometric analysis and full-text content coding to systematically examine the knowledge structure, technological evolution, application scenarios, technical capabilities, and experimental validation levels in this field. The aim is to identify key technological gaps during the transition of 3D mapping robots in constrained spaces from geometric mapping to semantic understanding, risk perception, and intelligent autonomous exploration, thereby providing guidance for future research directions and engineering deployment [18].

## 2. Methodology

**Figure 1:**
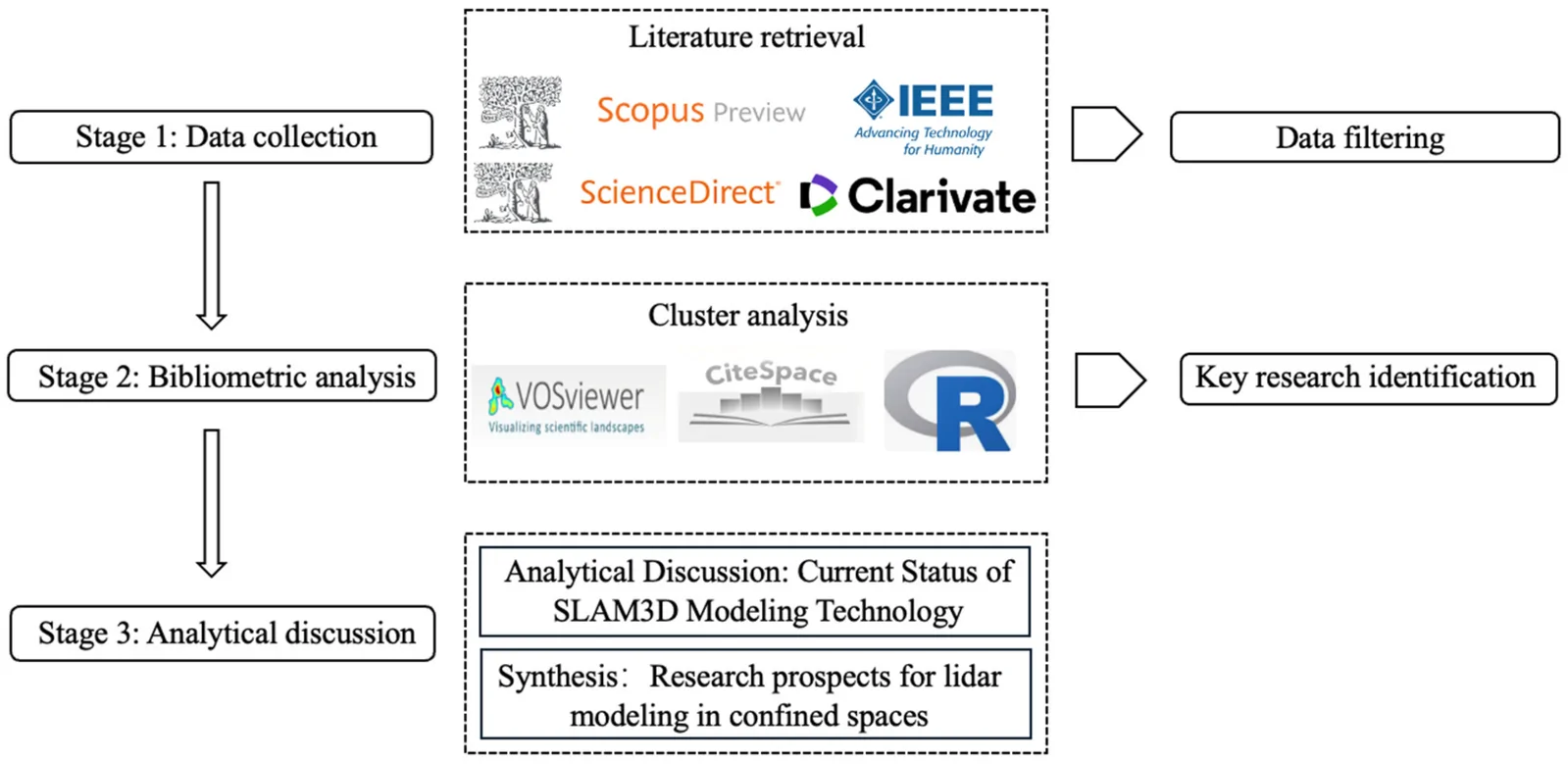
Multi-layered methodological structure underlying this systematic review.

Figure 1 illustrates the literature review process of this study. The first step is the literature search, which involves defining keywords related to the research direction using screening rules, and then conducting precise searches in four databases: Scopus, Science Direct, IEEE, and Web of Science. The second step involves using software such as VOSViewer, CiteSpace, and Bibliometrix to analyze the data, making the extensive literature content more visual. The third step is data analysis to draw conclusions, as well as to identify the development trends and research prospects of this research direction.

### 2.1. Data Collection

In conducting a literature review for a specific research domain, defining the search keywords constitutes the foundational step. Initially employing isolated terms such as “SLAM” results in an overly broad corpus of literature once the research scope—specifically, robot modeling within confined spaces—is established, thereby hindering the precise identification of relevant studies. Consequently, a structured search strategy involving multiple parallel keyword combinations was adopted. The keywords were categorized into three tiers for progressive searching, culminating in the following Boolean query: (“LiDAR SLAM” AND “confined environment”) AND (“topological mapping” OR “structure extraction”). Following the determination of these criteria, details of the specific search procedure are presented in Table 1.

To ensure methodological rigor and reproducibility, the complex literature screening process was visualized using a PRISMA flow diagram, adhering to standardized reporting guidelines.

**Figure 2:**
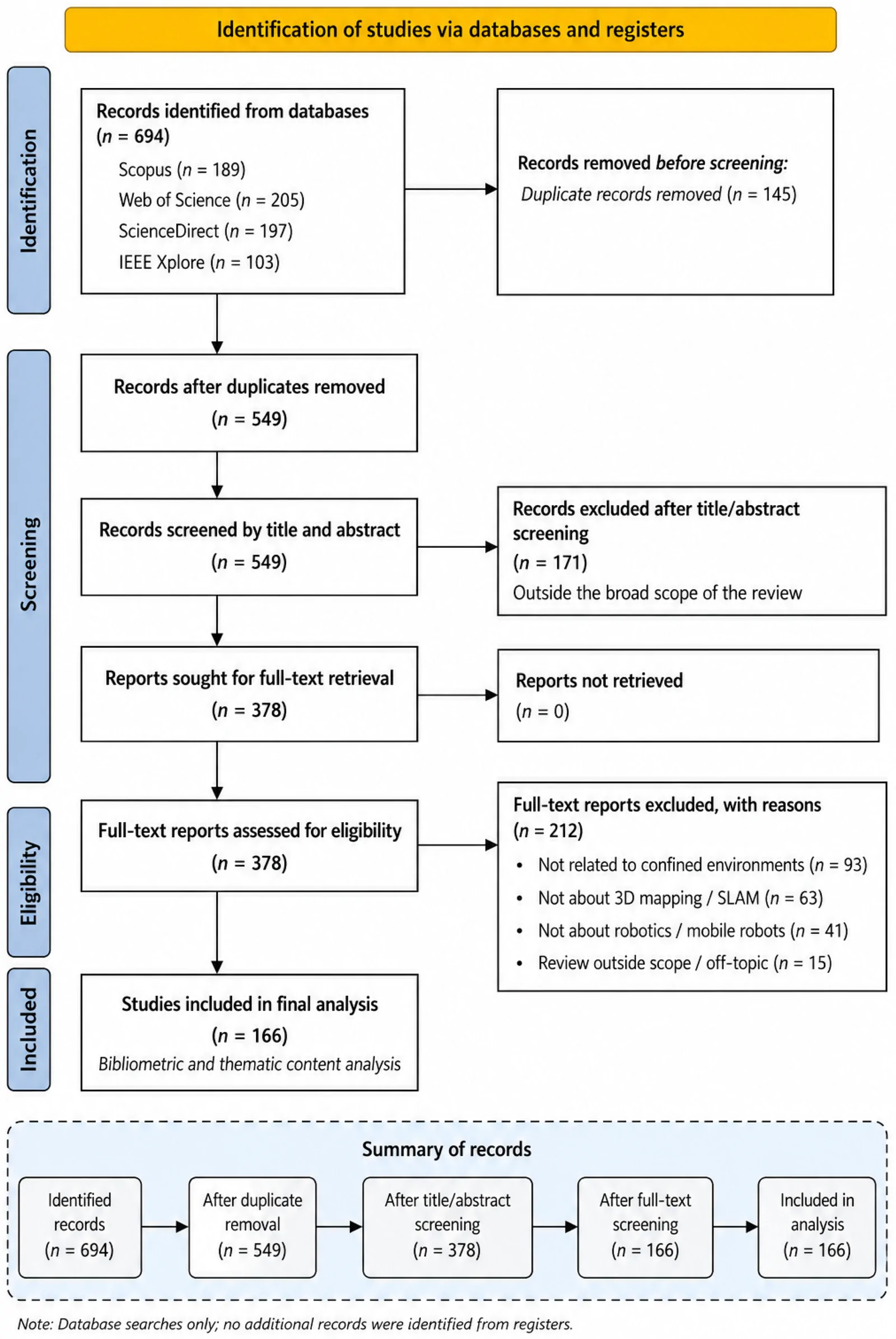
PRISMA flow diagram of database identification and study selection. Note: Searches of Scopus, Web of Science, ScienceDirect, and IEEE Xplore identified 694 records. After removing 145 duplicates, 549 records underwent title and abstract screening, and 378 reports were assessed for full-text eligibility. Following the exclusion of 212 reports, 166 studies were included in the bibliometric and thematic content analyses.

This study conducted a systematic review of research findings in the field of mapping for robots operating in confined spaces, adhering to the literature screening and inclusion protocol aligned with the PRISMA (Preferred Reporting Items for Systematic Reviews and Meta-Analyses) guidelines. Figure 2 presents the PRISMA flow diagram for this literature review, which explicitly documents the data collection process. Given that certain authoritative databases may not comprehensively index studies from specialized repositories, a multi-database integrated search strategy was adopted. Initially, a standardized search was executed across three general databases: Scopus, Web of Science, and ScienceDirect. Subsequent manual screening revealed that a substantial number of articles on the engineering aspects of robots for confined space modeling indexed in Scopus were sourced from IEEE journals; accordingly, engineering-relevant IEEE journals were incorporated into the database portfolio. Ultimately, four databases were finalized for this study: Scopus, Web of Science, ScienceDirect, and IEEE. A preliminary search across these four pre-specified databases yielded 694 articles. Duplicate entries across databases were then identified and eliminated, resulting in 549 unique articles proceeding to the subsequent screening phase.

In the initial screening stage, the relevance of the 549 articles was evaluated based on their titles, abstracts, and keywords, leading to the exclusion of 171 articles with low thematic relevance or those explicitly falling outside the research scope. Following this stage, 378 articles advanced to the full-text retrieval and in-depth assessment phase.

During the full-text eligibility review, the 378 articles were further analyzed with respect to their research objects, technical approaches, application scenarios, and research contributions. A total of 212 articles were excluded in accordance with pre-defined inclusion criteria. Ultimately, 166 highly relevant articles that met all inclusion criteria were selected for subsequent systematic review and bibliometric analysis. Figure 2 documents the selection process and the numbers of records retained at each stage. Furthermore, the process reflects the standardization and transparency of literature collection and screening procedures, thereby enhancing the credibility and academic value of the research outcomes. The 166 studies constitute the dataset for the bibliometric and thematic content analysis of this review; the reference list includes references directly cited in the main text, and may also include methodological literature and recent studies used in supplementary discussions. The complete list of the 166 included studies is provided in Table S1 of the Supplementary Materials.

Therefore, the total number of references is not required to match the number of studies in the analysis sample.

The literature search for this review was confined to Web of Science, Scopus, IEEE Xplore, and ScienceDirect, with no systematic retrieval conducted from preprint repositories. Consequently, studies that have not yet been indexed by these databases and are disseminated exclusively as preprints may have been omitted. This restriction in source coverage could diminish the extent to which the sample reflects the latest technological advancements; the potential implications of this limitation are further addressed in the Section 5.3.

### 2.2. Literature Analysis Tools

In bibliometric analysis, VOSviewer (version 1.6.20), CiteSpace (version 6.3.1), and Bibliometrix (version 4.5.3) currently stand as the most widely utilized and widely recognized tools within the domain of literature review. Each of these three tools possesses distinct advantages, encompassing functions such as network visualization, research trend identification, and statistical analysis.

VOSviewer is a specialized tool dedicated to the visualization of bibliometric networks, which can explicitly illustrate expressive structural relationships including keyword co-occurrence networks, international collaboration networks, and literature citation analysis networks. VOSviewer places particular emphasis on the representational capacity of large-scale knowledge network visualization, rendering it highly suitable for the visual analysis of complex databases.

CiteSpace ranks among the most representative tools in the fields of frontier identification and scientific knowledge evolution analysis. CiteSpace was used to visualize keyword clusters and their temporal relationships. Notably, its timeline visualization function can effectively demonstrate the occurrence density of target keywords across different time intervals, as well as their associative relationships with other keywords along the temporal development trajectory.

Bibliometrix is an open-source bibliometric analysis toolkit developed based on the R programming language. It is designed to deliver a comprehensive scientific mapping workflow, integrating flexibility, reproducibility, and robust statistical analysis capabilities. It enables the visualization of the temporal evolution of keyword associations and supports the statistical quantification of publication outputs from different countries in specific research domains within defined timeframes, with results presented in a visual format.

Table 2 provides a comparative analysis of the strengths and limitations of the three bibliometric analysis tools employed in this study. As evidenced by the table, each tool exhibits distinct focus areas, and their complementary nature renders them well-suited for the analysis of literature data aggregated from multiple databases. Furthermore, the integration of these three tools enhances the comprehensiveness of research findings. Collaborative analysis using multiple software has emerged as the prevailing approach in contemporary systematic literature reviews and scient metric research.

## 3. Structural Analysis and Discussion

### 3.1. Annual Analysis of Literature

We analyzed annual publication counts, cumulative citations, and publication-year-specific h-indices for the 166 included studies published between 2016 and 2026. These metrics collectively reflect the evolutionary trajectory, academic activity, and changing influence of this research field over the past decade.

In this study, annual publication volume serves to illustrate the scale of research output and the pace of research progress, thereby indicating the level of activity within the field. Total citation counts denote the academic influence and recognition of existing research findings in subsequent studies. As a key indicator, the h-index integrates insights from the above two metrics to assess the overall level of academic contribution. By analyzing the 166 selected papers using these metrics, we can identify the formation and diffusion processes of research hotspots in domains such as SLAM, 3D technologies, and confined spaces.

**Figure 3:**
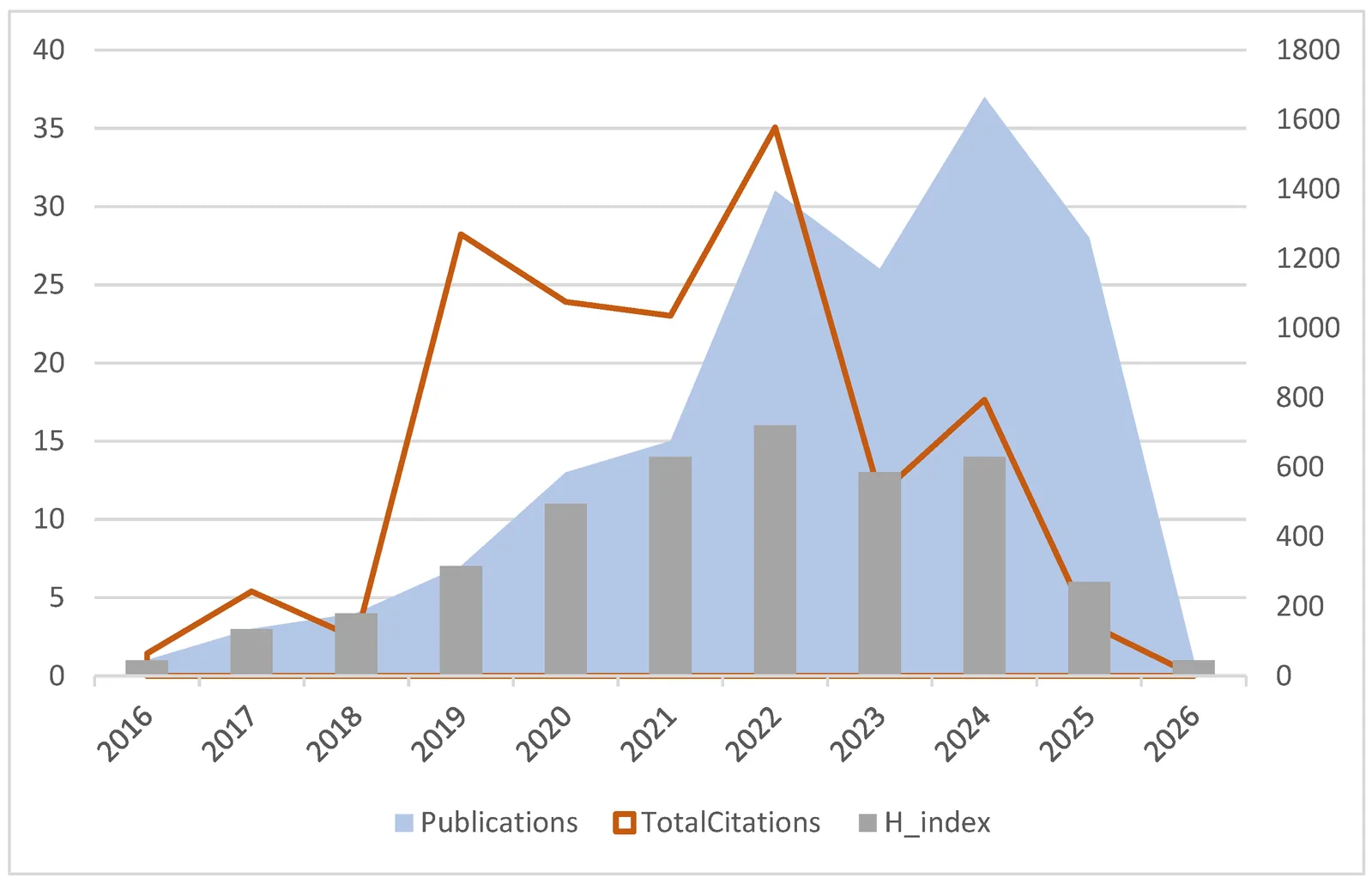
Annual Publications, Citations, and H-index Statistics (2016–2026).

Figure 3 presents the annual citation counts and h-index derived from the 166 studies included in this review. The steady increase in publication volume from 2019 to 2022 indicates a gradual expansion of research coverage within the sample corpus. Although subsequent years exhibited fluctuations, publication rates remained higher than those in earlier periods, reflecting sustained research activity and non-linear growth in annual output.

Publication counts and citation indicators followed different patterns. While 2024 recorded the highest number of publications, the cohort of papers published in 2022 achieved the peak cumulative citations and h-index. This discrepancy suggests that a larger quantity of publications does not necessarily correlate with greater citation influence. Furthermore, the high total citation count for the 2019 cohort was significantly driven by highly cited foundational works such as RTAB-Map [19], underscoring the substantial impact that a few widely adopted seminal studies can have on the overall citation distribution.

The relatively low citation metrics observed in recent years may primarily be attributed to the limited time available for citation accumulation, rather than indicating a decline in research quality or a transition into an adjustment phase. Similarly, given the small sample size for 2026, these data are insufficient for robust trend inference. Consequently, these results should be interpreted as reflections of the temporal distribution and citation performance of the included literature. Definitive conclusions regarding technological maturity and shifts in research directions require further analysis integrated with subsequent sections.

### 3.2. Citation Impact Analysis

Table 3 and Table 4 present citation metrics for 166 publications in the field of 3D modeling robots in confined spaces. All papers are ranked and analyzed based on citation counts, with a comprehensive evaluation incorporating both total citations and average annual citations. Compared to relying solely on total citation numbers, incorporating average annual citations provides a more accurate reflection of recent impact and research trends, making this approach widely adopted in bibliometric studies.

Comparing Table 3 and Table 4 reveals that high-impact publications are primarily concentrated between 2019 and 2024, indicating rapid development in this field in recent years—consistent with earlier H-index analysis results. Meanwhile, a research framework centered on LiDAR SLAM, multi-sensor fusion, robotic scanning, and data acquisition in extreme environments has gradually taken shape.

In terms of total citations, “RTAB-Map: An Open-Source LiDAR and Visual SLAM Library for Large-Scale and Long-Term Online Operation” ranks first with 883 citations, demonstrating its status as a foundational contribution in the field. “A Comparative Analysis of LiDAR SLAM-Based Indoor Navigation for Autonomous Vehicles” [20] and “M2DGR: A Multi-Sensor and Multi-Scenario SLAM Dataset for Ground Robots” rank second and third, respectively, highlighting the importance of SLAM performance evaluation, multi-scenario applications, and dataset construction as key foundations in current research.

Regarding average annual citations, “Present and Future of SLAM in Extreme Environments: The DARPA SubT Challenge” and “Swarm-SLAM” [21,22] achieve 53 and 42 citations per year, respectively, reflecting that SLAM in extreme environments, multi-robot collaboration, and autonomous exploration have become major research hotspots in recent years. Compared to total citations, average annual citations better capture the latest academic influence of publications.

Further cross-analysis shows that 17 out of the top 20 papers in both rankings overlap, indicating sustained and stable influence among core literature. By publication year, the top 20 papers by total citations peak in 2020 (30%), while those by average annual citations peak in 2022 (35%), suggesting that 2020–2022 was a critical period of rapid advancement in the field.

Overall, Table 2 and Table 3 reveal the knowledge evolution trajectory of this domain: early research focused primarily on SLAM algorithms and LiDAR-based localization and mapping, then progressively advanced to multi-sensor fusion, and further expanded into autonomous exploration and robot collaboration in extreme environments, offering valuable guidance for future research directions.

### 3.3. Co-Occurrence Network Analysis of Key Words

This figure presents a keyword co-occurrence network generated using VOSviewer based on an analysis of 166 scholarly publications. Keyword co-occurrence analysis—a foundational text-mining methodology—identifies thematic relationships and maps the conceptual architecture of a research domain by quantifying the frequency with which pairs of keywords co-appear within individual documents. 

**Figure 4:**
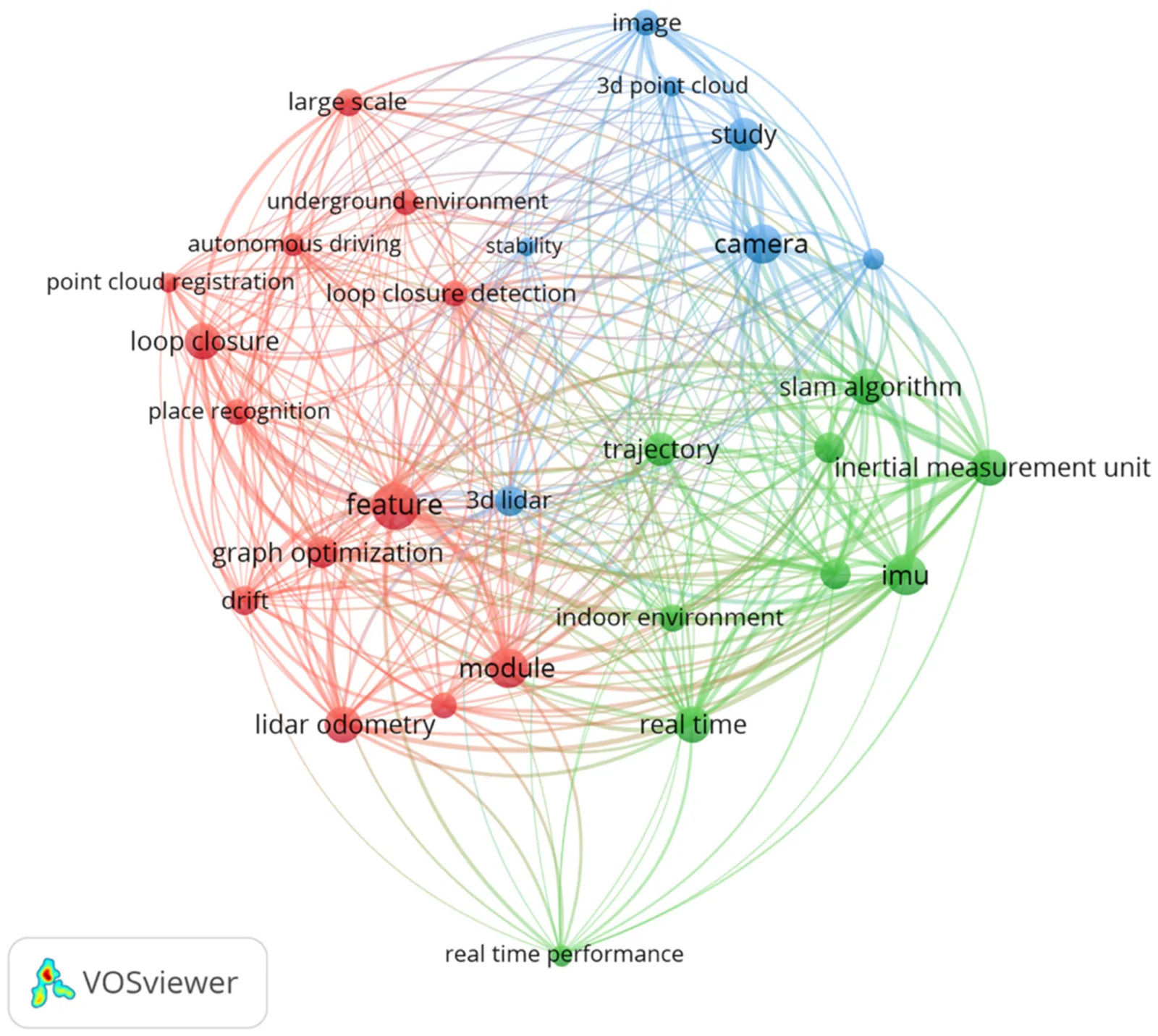
VOSviewer keyword co-occurrence map. The red cluster represents SLAM optimization, the green cluster represents real-time localization and multi-sensor fusion, and the blue cluster represents visual perception and 3D reconstruction.

As shown in Figure 4, the network comprises three principal thematic clusters centers on SLAM optimization; the green cluster addresses real-time localization and multi-sensor fusion; and the blue cluster pertains to visual perception and 3D reconstruction. SLAM occupies a structurally central position in the network, exhibiting strong bibliometric linkages with terms including “graph optimization”, “loop closure detection”, “LiDAR odometry”, “camera”, and “inertial measurement unit (IMU)”. This centrality affirms SLAM’s role as the foundational technological framework for robotic 3D modeling in confined spaces. The prominence of “graph optimization” and “loop closure detection” [23,24,25,26,27,28,29] reflects a methodological maturation—from basic mapping toward high-precision, high-robustness SLAM systems capable of mitigating drift accumulation in prolonged operation within constrained environments [30,31,32,33].

The red cluster predominantly encompasses laser-based SLAM research conducted in geotechnically complex and spatially constrained settings—including underground mines, transportation tunnels, and natural caves—thereby underscoring the expansion of SLAM applications into domain-specific operational contexts. The green cluster highlights the increasing adoption of heterogeneous sensor fusion, particularly integrating IMUs with LiDAR and vision modalities, as a robust strategy to enhance pose estimation accuracy and field deployability. The blue cluster signals a substantive conceptual transition: from geometric map generation toward semantic scene understanding, object-level reasoning, and context-aware autonomous decision-making. 

Collectively, the network demonstrates high node connectivity density, indicative of pronounced interdisciplinary convergence—spanning robotics, computer vision, sensor engineering, and geospatial informatics. Moreover, it evidences a sustained epistemological shift in the field: from data acquisition and geometric representation toward intelligent perception, semantic interpretation, and adaptive autonomy—capabilities essential for deploying robotic systems in extreme or mission-critical confined-space scenarios.

### 3.4. Keyword Clustering Analysis

CiteSpace software (version 6.3.1) incorporates a keyword clustering analysis function, which not only identifies the core structure of a research field but also clarifies the interrelationships between different research topics. Additionally, it enables the analysis of potential overlaps in research hotspots and facilitates the prediction of future development trends within the field.

**Figure 5:**
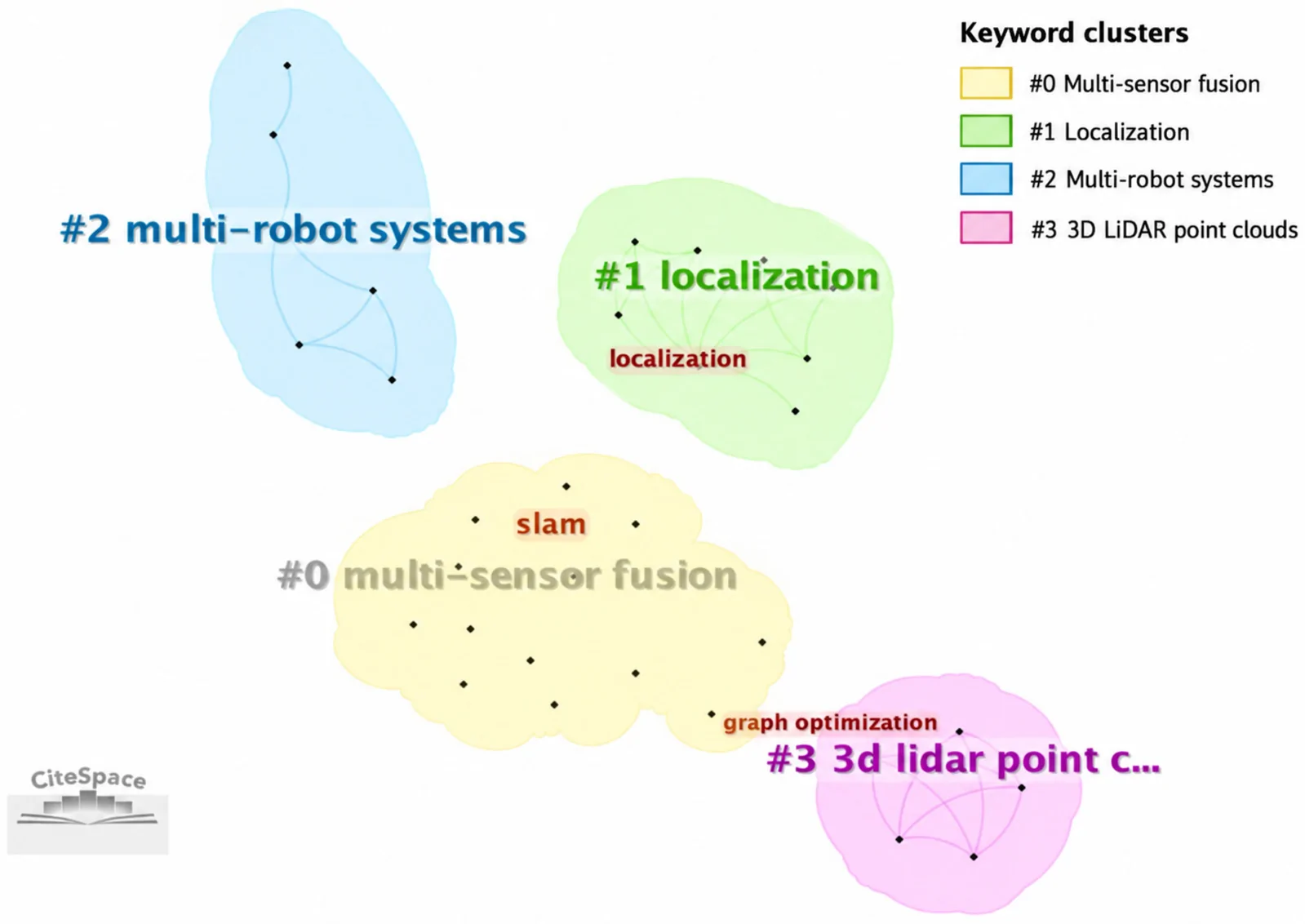
Keyword Clustering Cloud Map.

Figure 5 presents a keyword-based clustering cloud map generated from bibliometric analysis of 166 peer-reviewed publications. It reveals a cohesive technical architecture centered on Simultaneous Localization and Mapping (SLAM), with integrative linkages to multi-sensor fusion, autonomous localization, multi-robot coordination, and 3D LiDAR point cloud modeling. This architecture reflects three convergent scholarly trends: interdisciplinary convergence across robotics, perception engineering, and geospatial informatics; methodological advancement toward intelligent, adaptive systems; and systemic evolution toward collaborative operational paradigms.

The analysis yields four principal thematic clusters: #0 Multi-Sensor Fusion, #1 Autonomous Localization, #2 Multi-Robot Coordination, and #3 3D LiDAR Point Cloud Modeling. Cluster #0 exhibits the highest node density and centrality, confirming multi-sensor fusion as the foundational research theme across the corpus. Confined-space environments—including underground mines, transportation tunnels, natural caves, and post-disaster rubble fields—are characterized by severe perceptual constraints: low illumination, spatial confinement, occlusion-rich topologies, and dynamic structural uncertainty [34,35,36,37,38]. Under such conditions, single-modality sensing proves insufficient for reliable state estimation [39,40,41,42]. Consequently, the synergistic integration of LiDAR, visual, and inertial measurement unit (IMU) data has become an indispensable methodological pillar—enabling enhanced geometric fidelity, temporal consistency, and system-level robustness [14,43,44,45,46,47,48,49,50,51,52,53,54,55,56,57,58].

1. Cluster #1 demonstrates high internal cohesion and strong bibliometric coupling with SLAM-related terms (e.g., loop closure, pose graph optimization, trajectory estimation), affirming autonomous localization as a persistent core research priority [59,60,61,62,63,64,65,66,67,68]. In GNSS-denied environments, robots must achieve centimeter-level pose accuracy while concurrently constructing spatial representations of their surroundings. When GPS is available, a GPS–LiDAR fusion route exists, but this condition cannot be directly transferred to an underground environment where GNSS is unavailable throughout the entire route [69,70,71,72]. Thus, localization in this domain transcends mere coordinate determination—it constitutes a tightly coupled process of real-time state estimation and environmental mapping [73,74,75,76,77,78,79].
2. Cluster #2 displays structural autonomy within the network, indicating that multi-robot coordination has matured into a distinct yet still emergent research domain. Its applications—such as distributed exploration, task partitioning, and consensus-based mapping—offer demonstrable advantages in coverage efficiency, operational scalability, and fault resilience [80,81]. However, its relatively weak inter-cluster connectivity with #0 (multi-sensor fusion) and #1 (localization) suggests limited cross-thematic integration, pointing to a critical opportunity for future work on unified frameworks for heterogeneous multi-robot SLAM and collaborative sensor fusion.
3. Cluster #3, though modest in size, exhibits exceptional thematic specificity and conceptual coherence, underscoring 3D LiDAR point clouds as a primary representational modality. As a dense, metrically accurate, and sensor-agnostic spatial descriptor, point cloud data provide the foundational substrate for geometric reconstruction, semantic segmentation, change detection, and physics-informed navigation planning—thereby enabling a transition from geometric mapping to semantic-enriched, decision-ready environmental models [82,83,84,85,86,87,88,89,90,91].

Collectively, the clustering structure evidence a well-established technical paradigm: SLAM serves as the algorithmic nucleus; multi-sensor fusion provides the perceptual substrate; 3D point cloud modeling delivers the geometric and semantic representation layer; and multi-robot coordination constitutes the emerging operational abstraction. This paradigm is now advancing toward higher-order capabilities—including context-aware perception, long-horizon autonomous exploration under uncertainty, and resilient, adaptive multi-agent coordination—essential for deploying robotic systems in mission-critical confined-space scenarios.

### 3.5. Time Evolution and Timeline Analysis

To further explore the evolutionary process and future development trends in the field of modeling robots for confined spaces, this study conducts an analysis using a keyword clustering timeline map generated by CiteSpace software, complemented by a bubble chart of annual frequency evolution of keywords analyzed via bibliometric code. The integration of these two visualizations enables mutual complementarity, thereby comprehensively reflecting the formation process, evolutionary path of research themes, and the changing trends of research hotspots in recent years.

**Figure 6:**
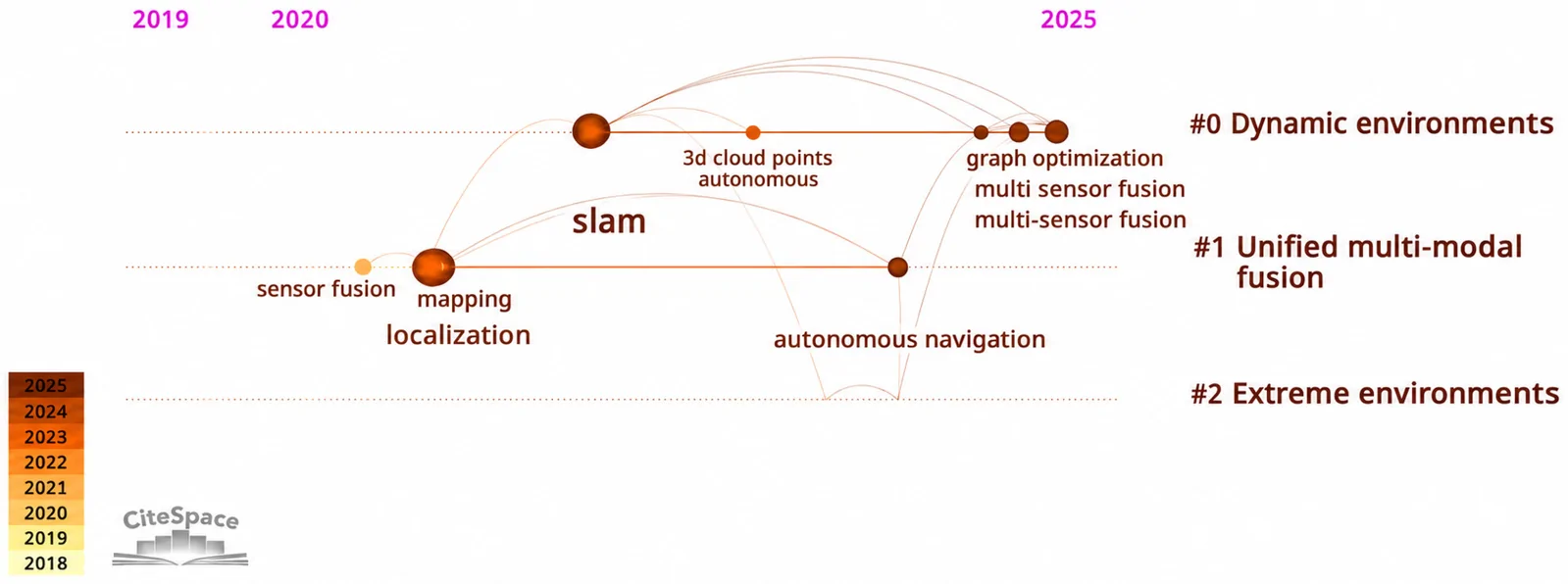
CiteSpace Timeline.

Based on the timeline graph shown in Figure 6, the research in the field of 3D modeling robots for confined spaces demonstrates a distinct evolutionary trajectory, which is specifically manifested in three key phases: fundamental localization and map construction, advancement of core SLAM technologies, and expansion of multi-sensor fusion and autonomous intelligent applications.

During the initial phase spanning 2018 to 2020, the primary research trend centered on foundational localization and modeling studies [92,93]. Notably, “localization” and “mapping” emerged as the earliest and most enduring research themes in this stage, which marked the technical infancy of the research direction. The core objective of this phase was to tackle the fundamental navigation problems of robots.

During the period around 2020–2022, the node corresponding to SLAM (Simultaneous Localization and Mapping) exhibited a marked increase in size and occupied a central position within the entire timeline network. Key characteristics of this phase include enlarged node dimensions, extended duration, and high-intensity connections with other research topics—collectively indicating that SLAM had emerged as a foundational technology in the field. This research domain thus transitioned from an early stage focused on single technical functions to a more mature phase characterized by systematic modeling development [94,95,96].

Since 2023, research hotspots in this field have trended toward intelligentization and general applicability across diverse extreme environments. As illustrated in the figure, multi-sensor fusion has emerged as one of the most active research foci in recent years—a phenomenon that reflects the growing complexity of application scenarios, where single-sensor solutions are no longer sufficient to meet sophisticated operational demands. Recent studies have leveraged multi-source information fusion to enhance the environmental perception capabilities of robots, with the aim of improving positioning accuracy, the completeness of 3D maps, and adaptability to varying environments [97,98]. This evidence indicates that the research direction is gradually advancing toward intelligentization.

**Figure 7:**
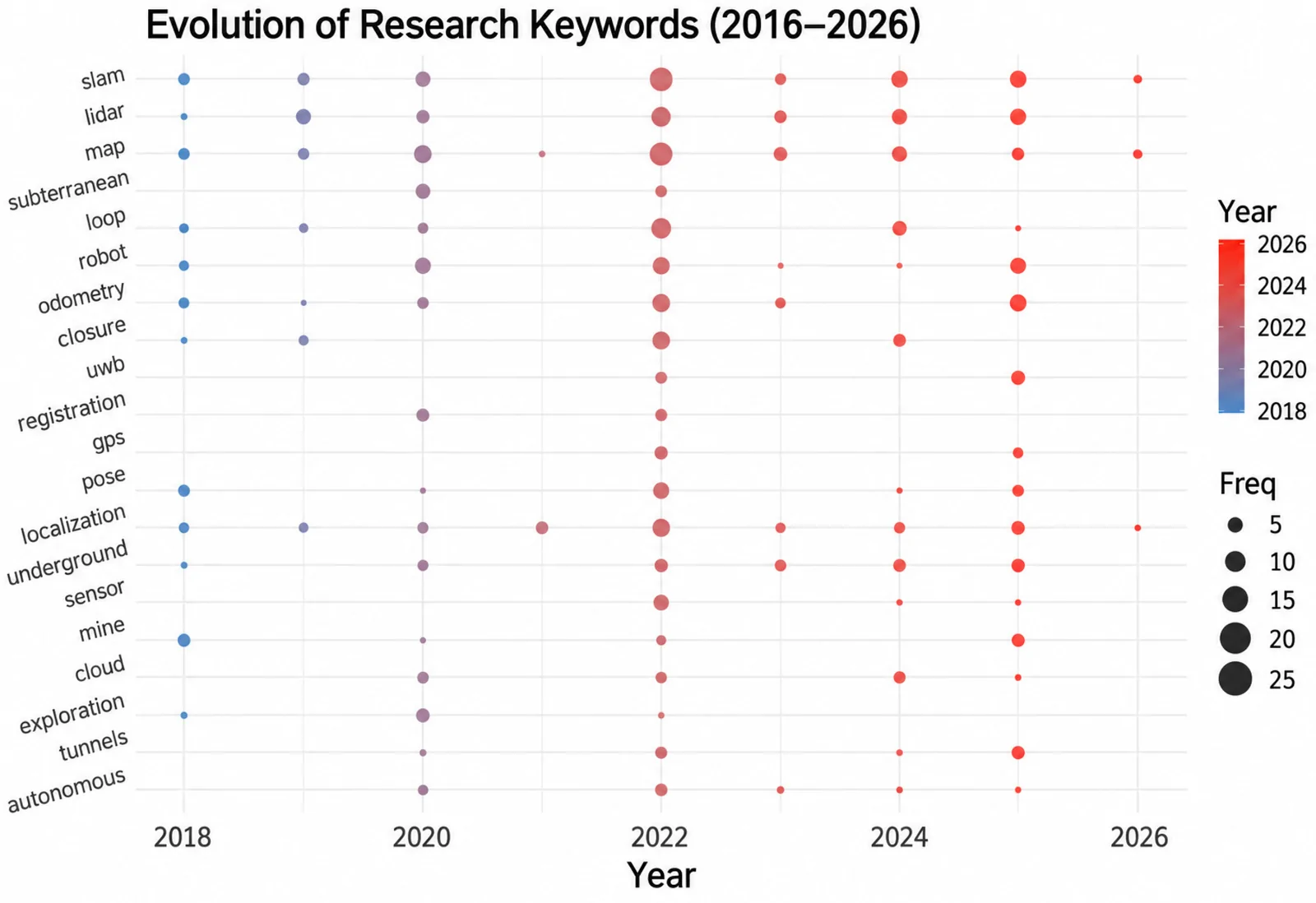
Annual Frequency Evolution Graph of Keywords in Research on 3D Modeling Robots for Confined Spaces.

Figure 7 presents the year-by-year variation analysis of keyword frequencies for 166 academic papers, generated via RStudio (version 2026.01.1+403) code. This figure is intended to complement and supplement the information provided by the CiteSpace timeline visualization presented earlier. As indicated by the prominence of the focal points in this graph, keywords including SLAM, LiDAR, map, localization, underground, tunnels, and autonomous maintain a high level of activity, suggesting that the research focus in this domain has shifted toward the practical implementation of solutions in specific scenarios.

When considering Figure 6 and Figure 7 collectively, it is evident that the current research on 3D modeling robots for confined spaces is underpinned by a developmental framework centered on SLAM, multi-sensor fusion, and autonomous navigation. Nevertheless, future research endeavors still necessitate advancements in several key areas—challenges that subsequent researchers are tasked with addressing. These include the highly irregular spatial states induced by post-disaster collapsed structures, path planning for multi-branched structures, extraction of spatial structural features, and subsequent risk assessment. Concurrently, these issues also form part of the source material for the problem statement within this research domain.

## 4. In-Depth Analysis of Research Landscape

The preceding three chapters constitute the most fundamental statistical analysis of literature information and the analysis of preliminary presentation results. After this, a more in-depth analysis of the literature is required to identify the most significant research gaps in the field. In the in-depth analysis, it is necessary to categorize and summarize the research fields and contents of these 166 articles. By integrating the basic analyses such as keywords, timelines, citation counts, and countries conducted earlier with the comprehensive analysis of research contents, and by analyzing from various perspectives, more in-depth and persuasive conclusions can be drawn, thereby leading to the subsequent research contents and research questions.

### 4.1. Application Scenario Analysis

**Figure 8:**
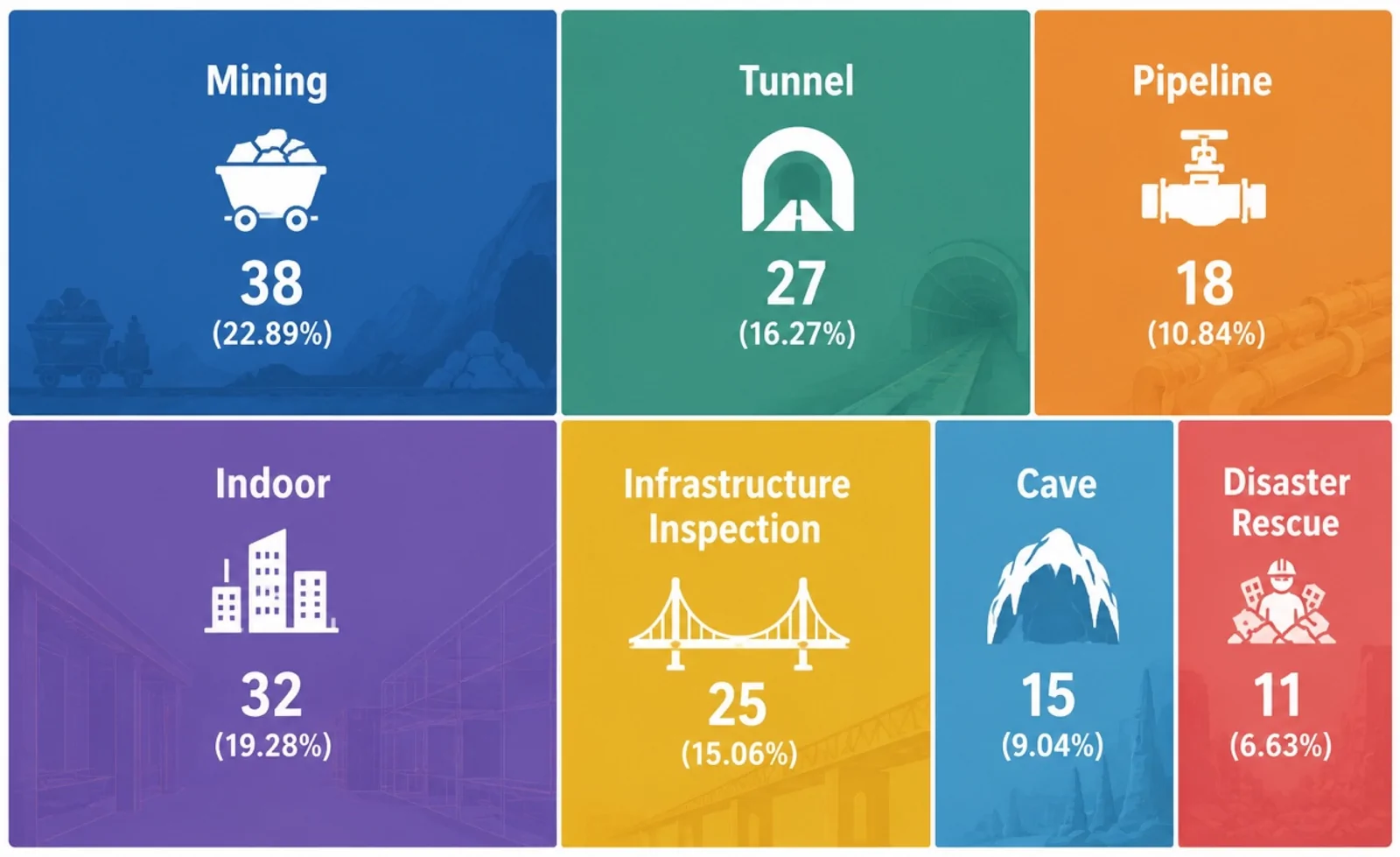
Research Domain Distribution of 166 Publications.

Figure 8 illustrates the classification of research domains extracted through content analysis of 166 analytical studies. In general, four application scenarios—Mining (22.89%), Indoor Environment (19.28%), Tunnel (16.27%), and Infrastructure Inspection (15.06%)—collectively account for 73.5% (122 out of 166) of all studies. This finding indicates that contemporary research predominantly focuses on spaces characterized by relatively regular spatial structures, well-defined or pre-understood overall configurations, and stable environmental features.

This phenomenon is characterized by explicit engineering demand-driven features. Spaces such as mines, underground tunnels, building interiors, and underground pipelines [99,100,101,102,103,104,105,106,107,108,109,110,111,112,113], which possess fixed spatial topologies and continuous regular geometric structures, serve as optimal platforms for constructing standardized experiments. They also provide the most efficient experimental environments for training functions of SLAM algorithms, including localization, loop closure detection, and map optimization. Additionally, most of these scenarios fall under mature industrial requirements; consequently, numerous countries and regions allocate significant research resources, rendering application research in this domain one of the most active segments within the broader scope of 3D spatial modeling [114,115,116,117].

Compared to regular industrial application scenarios, the two types of highly irregular and uncertain environments—cave geological research and post-disaster rescue—account for a very small proportion, which also indirectly indicates that research on these two areas is clearly insufficient [118].

This difference arises because non-regular environments have the following characteristics:

1. The spatial topology is unknown and highly dynamic, making it impossible to pre-map.
2. Channel widths vary significantly, with numerous branches, dead ends, and random obstacles.
3. Lighting conditions are extremely poor, with dust, smoke, and occlusions severely degrading visual sensor performance [119].
4. GNSS signals are completely unavailable, and communication links are prone to interruption.
5. The ground is unstable, posing risks of robot sinking, tipping, or getting trapped.

These practical factors impede the stable operation of early autonomous mobile robots integrated with SLAM technology and further escalate the technical difficulties in robot autonomous decision-making, exploration, and long-term deployment [120,121,122,123]. Thus, it can be concluded that current research still caters to spatial environments with relatively controllable conditions, while complex spaces that hold greater engineering value and application demand remain a gap in the current research landscape. From the perspective of the increasingly diverse demands of practical application scenarios in the future, non-structured environments will be the core challenge for the future development of robots. In future applications, as more human-performed tasks are transferred to AI and robots for execution, it will be necessary to endow robots with certain autonomous decision-making capabilities. This constitutes a primary research objective for the future and is also one of the most critical development trends revealed by literature analysis.

Overall, more than 70% of current research still focuses on structured environments, while studies on autonomous mapping, decision-making, and environmental cognition in unstructured settings remain significantly insufficient. This indicates that robots operating in confined spaces today primarily address the challenge of “how to map,” but have yet to adequately solve the problems of “how to understand the environment” and “how to act autonomously.” This imbalance in research precisely represents the key direction for future breakthroughs in intelligent robotics for confined spaces.

### 4.2. Country–Research Domain Analysis

**Figure 9:**
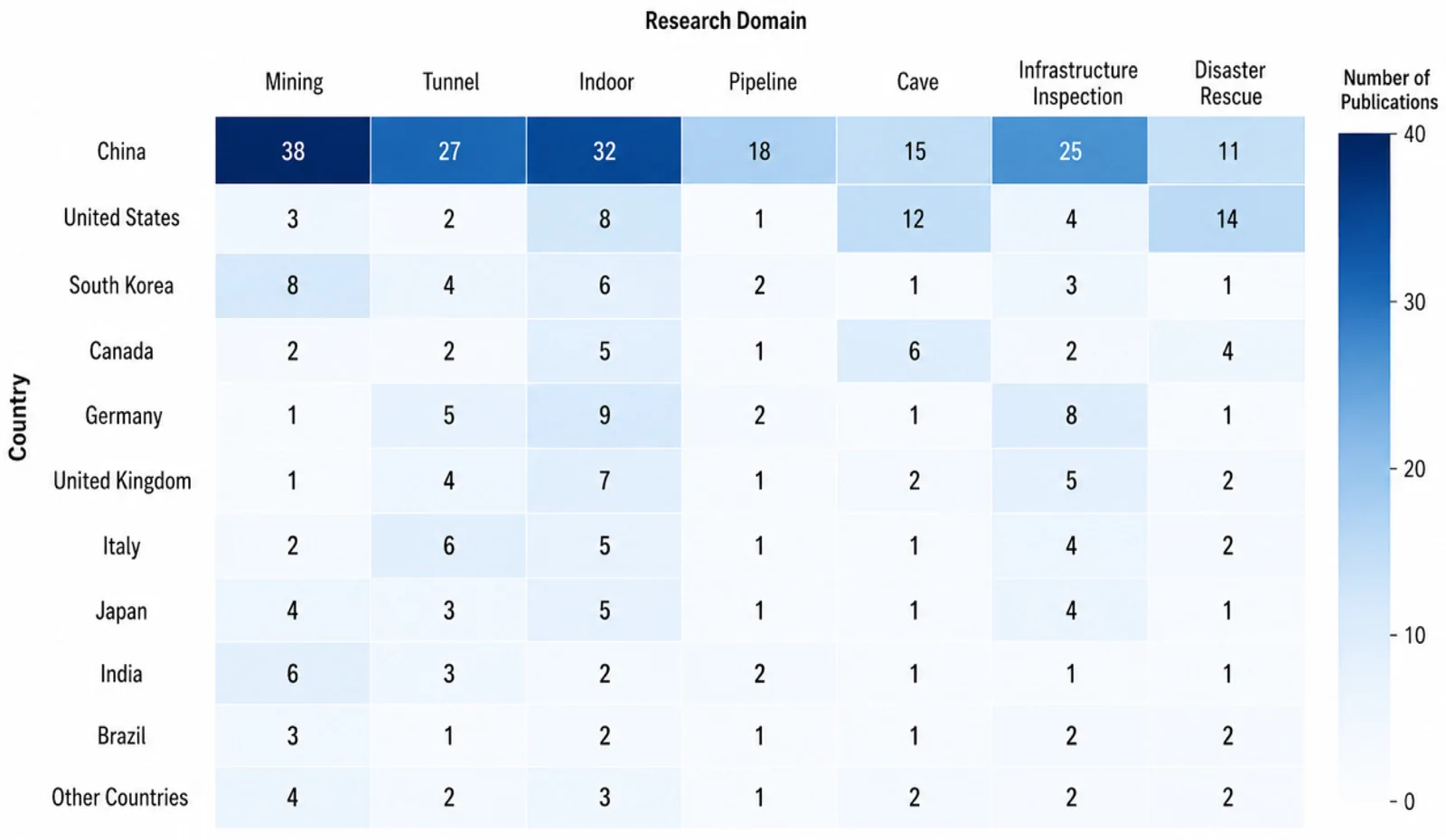
Country × research domain distribution of the 166 studies included in this review. Note: Cell values represent publication counts rather than percentages. Countries were identified from author affiliations using full counting: each publication was counted once for each affiliated country. Consequently, country counts may overlap and should not be interpreted as mutually exclusive shares of the 166 studies.

To further explore the landscape of narrow-space detection research across different countries, this study conducts an in-depth analysis using a Country × Research Domain Heatmap. Specifically, it investigates the research focuses of various countries within distinct application domains. In contrast to the country-specific publication volume analysis presented in Chapter 3, the analysis in this section not only delves deeper but also more effectively elucidates the correlation between research orientations and the demands of national industrial applications. Figure 9 shows the distribution of the 166 included studies across countries and research domains.

This figure uses the 166 included studies as the analytical sample, with country scope limited to the countries where the authors’ affiliated institutions are located, covering regions such as Asia, Europe, and the Americas, rather than a comprehensive count across all countries globally. Because multinational publications are counted once for each affiliated country, the sum of country-level publication counts may exceed 166. The analysis results reflect only the distribution of countries and research fields within this review’s sample.

Within the included sample, China accounts for the largest publication counts in several industrial application domains. This leadership stems from a confluence of factors—including the country’s geographically diverse terrain, extensive mineral resource endowments, and sustained, large-scale investment in urban and industrial infrastructure. To date, scholarly efforts have predominantly centered on three interrelated technical pillars: simultaneous localization and mapping (SLAM), robust multi-sensor fusion architectures, and autonomous inspection systems tailored for complex, large-scale industrial settings. Emerging trends indicate a strategic shift toward AI-driven decision support—integrating perception, reasoning, and adaptive control to enable higher levels of operational autonomy and intelligence. In contrast, research efforts in the United States are more concentrated on application scenarios such as cave exploration, underground space utilization, and post-disaster rescue operations. The focus lies on capabilities including autonomous exploration in uncharted environments, multi-robot coordination, and decision-making under communication-constrained conditions, rather than being confined to technical optimizations of traditional Simultaneous Localization and Mapping (SLAM) systems.

In contrast to fragmented development patterns observed elsewhere, European countries exhibit a relatively balanced research landscape, with scholarly efforts predominantly centered on indoor environment perception and infrastructure inspection scenarios. European research prioritizes three core technical dimensions: the long-term operational stability of robotic systems, human–robot collaborative interaction capabilities, and reliable deployment in real-world industrial contexts. This strategic focus has fostered a distinct engineering application-oriented research paradigm across the region. While Japan and South Korea exhibit relatively constrained publication output compared to leading research economies, their scholarly focus remains highly concentrated, with core investigations centered on indoor mapping, mobile robot navigation, and visual perception. Research emphases vary across the countries represented in the sample; the factors underlying these differences require further investigation. However, their overall publication output remains limited, and research themes are relatively concentrated—findings that suggest substantial untapped potential in the field of constrained-space robotics research.

Overall, research priorities across nations are strongly correlated with their industrial demand profiles, infrastructure development levels, and targeted application scenarios, giving rise to distinct regional specialization patterns. Currently, the field confronts persistent challenges including uneven regional development trajectories, skewed distribution of research scenarios, and gaps in intelligent autonomous decision-making capabilities. Nevertheless, these disparities also create avenues for cross-border collaboration and technological complementarity. Looking ahead, synergistic cooperation among countries is poised to drive confined-space exploration robots toward higher degrees of autonomy, intelligence, and engineering maturity.

### 4.3. Technology Capability Analysis

This section categorizes capabilities based on the research orientations of the included studies. The distinction between navigation and autonomous decision-making is defined as follows: **Navigation** is defined as the execution of path planning, trajectory tracking, or motion control toward a specified target; **Autonomous Decision-Making** is defined as the robot’s capacity to independently select exploration targets, adjust task strategies, or determine actions such as continuing, pausing, or returning, contingent upon mission objectives, environmental dynamics, or risk assessments. Notably, mere trajectory tracking or conventional local obstacle avoidance does not constitute task-level autonomous decision-making. However, studies satisfying the criteria for both categories are counted in each respective class.

**Figure 10:**
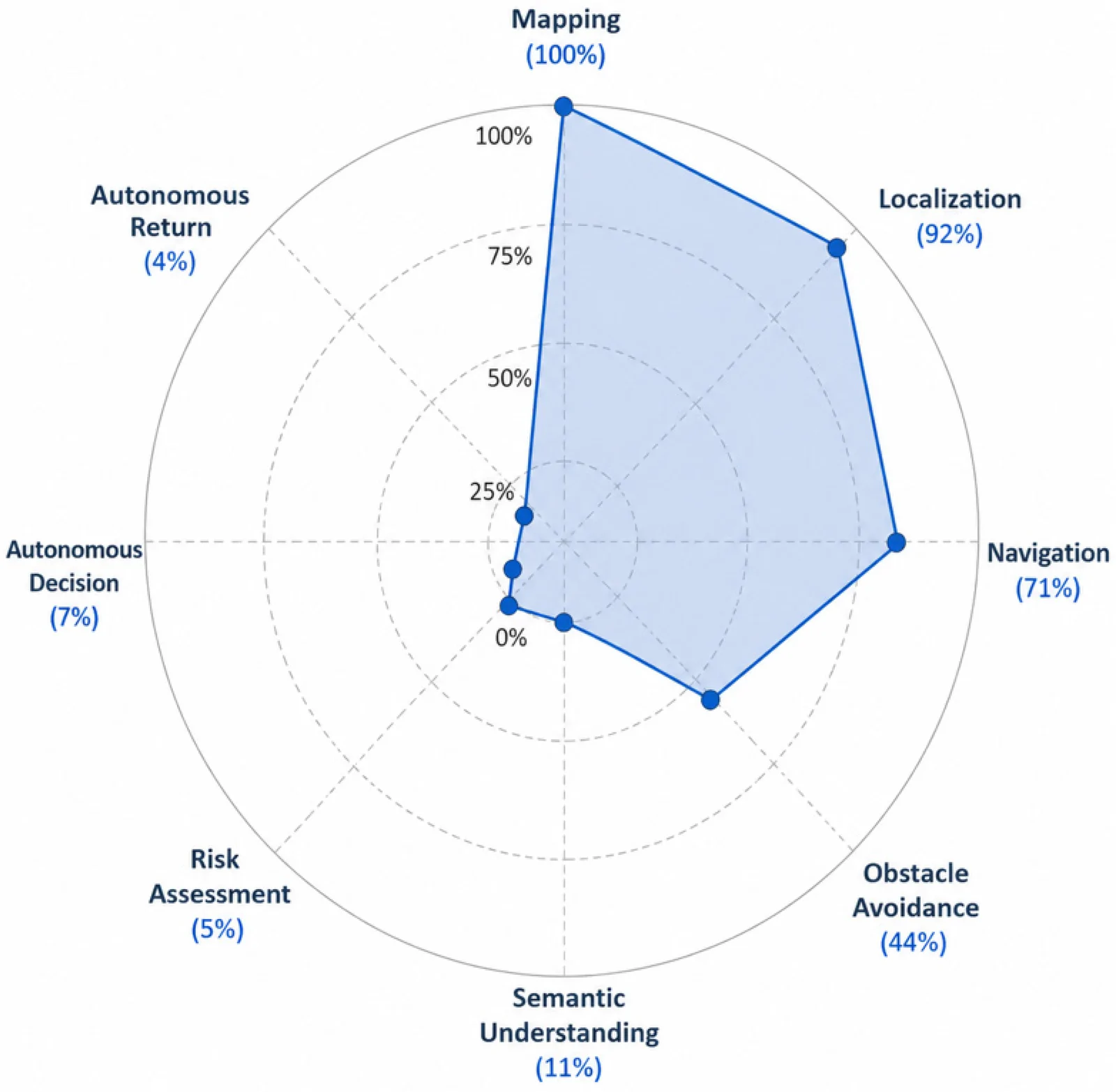
Technology Capability Distribution of Current Confined Space Modeling Robots.

The reported percentages indicate the proportion of the 166 included articles that explicitly investigate specific functionalities, rather than the prevalence of robotic systems demonstrating these capabilities. Given that individual studies may address multiple functionalities, the sum of category percentages does not necessarily equate to 100%. Consequently, these metrics reflect the distribution of research focus within the sampled literature and do not directly represent the overall technological maturity or capability levels across the entire field. Figure 10 shows the percentages of the 166 included studies that explicitly investigate each capability.

As can be concluded from the figure, the comprehensive focus of current technical research on narrow spaces still centers on map construction and localization functions, accounting for 100% and 92% respectively among the 166 analyzed articles—a notably high proportion. This indicates that nearly all studies are conducted around these two themes, which also reflects the inevitability of SLAM (Simultaneous Localization and Mapping) technology emerging as the core content of this research field. The proportion of the “Navigation” category in the figure reaches 71%, which indirectly demonstrates that in recent years, technological development has been oriented toward the navigation function of robots. Researchers have begun to focus on the autonomous movement capability of robots, thus promoting the evolution of robots from controlled mapping to autonomous navigation-based mapping [124,125,126].

However, when examining research focused on endowing robots with more intelligent capabilities, the proportion decreases significantly. The category of *Obstacle Avoidance* accounts for a mere 44%, indicating that while robots have acquired basic navigation functions, substantial research gaps persist in obstacle avoidance, route adjustment, and navigation in unknown environments—particularly in underground or irregular spaces. Even lower proportions are observed for Semantic Understanding (11%), Autonomous Decision-Making (7%) [127,128,129], Risk Assessment (5%), and Autonomous Return (4%). These underrepresented research areas highlight current limitations in the field, reflecting that most studies remain confined to the stage of spatial mapping, with robots yet to achieve genuine environmental understanding and analysis. Table 5 summarizes the research focus of the represented countries across mapping, perception, and intelligence.

The table above outlines the technological layout of different countries in this research field, where technologies are categorized into three major domains—mapping, perception, and intelligence—with a statistical summary of each country’s technical orientations. The findings in the table reveal distinct differentiating characteristics across various countries. China demonstrates notable activity primarily in the domains of Mapping and Perception, with research concentrated on foundational technologies including SLAM, 3D mapping, LiDAR-based perception, and multi-sensor fusion—reflecting a strong inclination toward industrial applications. In contrast, the United States exhibits a distinct advantage in the Intelligence domain, where its research prioritizes more advanced intelligent technologies such as autonomous robot exploration, multi-robot collaboration, and autonomous decision-making capabilities. European countries like Germany and the United Kingdom have pursued relatively balanced development, with a greater focus on system reliability and industrial applicability. Meanwhile, Japan and South Korea primarily direct their efforts toward application scenarios in manufacturing, such as indoor navigation and visual perception.

To summarize, the global research field of confined-space modeling robotics has established a relatively mature technical system in the domains of mapping and perception. Intelligence represents both the future developmental direction and a current technological bottleneck, with substantial research gaps remaining particularly in enabling robots to acquire advanced capabilities such as environmental understanding, autonomous decision-making, and risk assessment. Recent research has begun to extend the scope of robotic perception in confined spaces from geometric reconstruction to environmental interaction and local risk quantification. Soft-growing robots, which integrate gyroscopes, encoders, and flexible proximity sensing, leverage morphological adaptation and contact-guided navigation for exploration in unknown environments. While these studies demonstrate concrete progress in autonomous exploration and safety-aware perception, contact-based guidance and deformation alerts remain insufficient substitutes for task-level autonomous decision-making, structural collapse prediction, or autonomous evacuation protocols [130,131].

### 4.4. Research Maturity Analysis

To further assess the current technological maturity of the research, the experimental validation methods employed in these 166 papers were reorganized and analyzed. The experimental approaches were broadly classified into five stages: (1) Simulation Validation, defined as testing conducted exclusively within virtual environments; (2) Laboratory Validation, involving physical experiments performed in controlled laboratory settings or artificially constructed infrastructures; (3) Semi-Real Environment Validation, referring to evaluations carried out in test facilities or simulated scenarios that exhibit real-world engineering characteristics, though environmental and task conditions remain significantly constrained; (4) Field Validation, denoting on-site testing conducted within actual target operational scenarios; and (5) Commercial Deployment, which requires verifiable evidence of practical commercial implementation rather than mere theoretical applicability.

**Figure 11:**
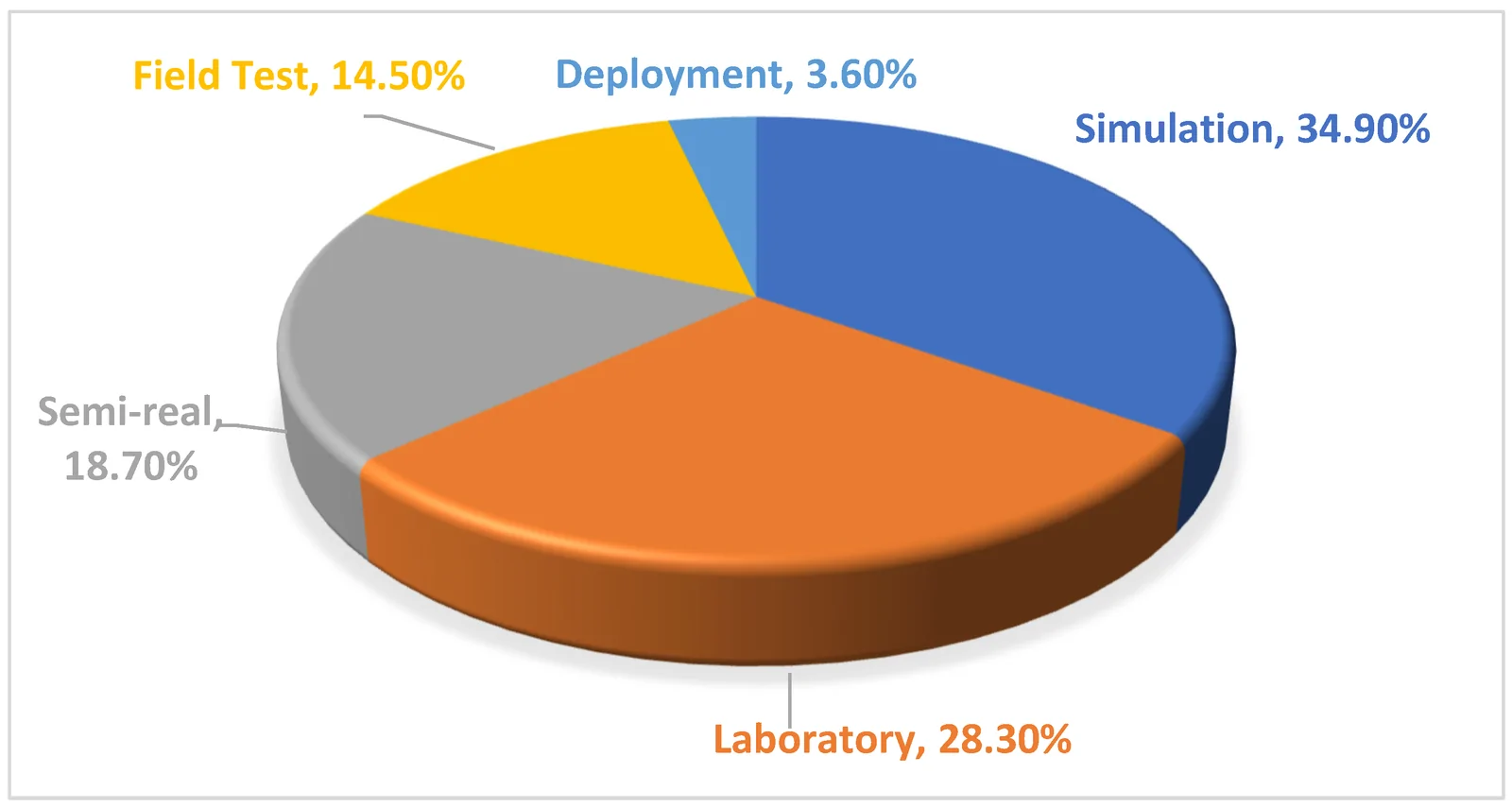
Distribution of Research Verification Maturity for Confined-Space Modeling Robots.

When a single study employs multiple validation methods, it is assigned to the highest tier supported by explicit empirical evidence. The distribution percentages are calculated relative to the baseline of 166 included articles. This classification describes the reported validation setting rather than methodological quality or system reliability. Their respective proportions were calculated, as illustrated in Figure 11.

Overall, simulation (34.90%) and laboratory (28.30%) together account for 63.20%, indicating that most current research remains in the stage of algorithm validation and lab testing. Although such studies offer advantages including low experimental cost, strong environmental controllability, and high reproducibility, which contribute to the rapid development of key technologies such as laser-based simultaneous localization and mapping (SLAM), multi-sensor fusion, and autonomous navigation, these evaluations do not, on their own, establish performance in complex real-world confined environments [132,133,134].

In contrast, the proportions of Semi-real Environment (18.70%), Field Test (14.50%), and Commercial Deployment (3.60%) are relatively low, indicating that current research is predominantly focused on small-scale spaces and still faces a substantial gap from large-scale engineering applications. Real-world confined spaces are characterized by communication constraints, signal loss, and complex obstacle-laden terrain, which pose significant challenges to the operational capabilities of robots—these challenges serve as a catalyst for technological advancement. Future research should further emphasize experimental validation in real-world scenarios, as well as investigations into key technologies for engineering deployment and autonomous decision-making, to facilitate the further development of related technologies [135,136,137,138,139,140,141].

### 4.5. Technology Evolution Framework

**Figure 12:**
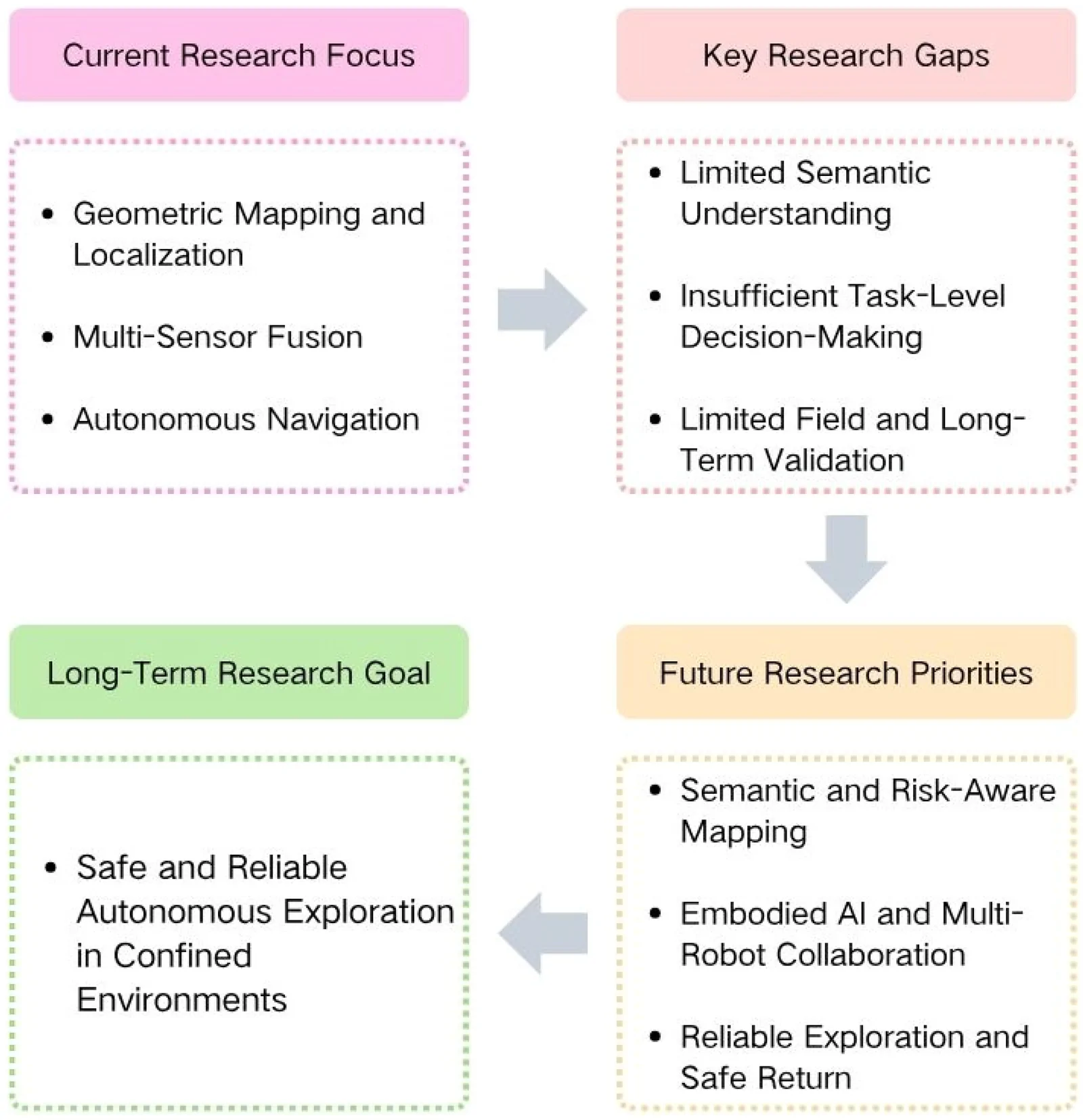
Technology Evolution Framework of Confined-Space Mapping Robots.

Figure 12 synthesizes multi-dimensional analytical findings, including keyword clustering, timeline analysis, technological development trajectory, and research maturity assessment, thereby systematically summarizing the technological evolution within the target research domain. As illustrated in the figure, the technological advancement of this field is undergoing a transition from geometric mapping to intelligent autonomy. Early research efforts were predominantly centered on geometric modeling, leveraging foundational mapping technologies such as SLAM, with the core objective of establishing spatial information representation in unknown environments. With the progression of multi-sensor fusion techniques, the research focus has gradually shifted from basic information acquisition to intelligent environmental comprehension.

Current research is progressively moving toward intelligent development. In recent years, AI technology has advanced rapidly and permeated diverse industries, with AI robotics emerging as a high-profile cutting-edge technology. This implies that robots within this research domain are required not only to fulfill spatial modeling tasks but also to possess capabilities in environmental understanding and autonomous decision-making. As depicted in the figure, current research remains at the stage of geometric perception and autonomous navigation. With the increasing complexity of application environments, the demand for robot intelligence continues to escalate. Thus, future research should further facilitate the deep integration of artificial intelligence and robotic systems, enabling a technological leap for confined-space robots from “Perception-Oriented Mapping” to “Intelligence-Oriented Autonomous Exploration.”

### 4.6. Three-Dimensional Analytical Framework

To enable the three-dimensional analytical framework to be applied in specific case comparisons, this paper establishes operational definitions and grading criteria for scenario complexity, autonomy capability, and deployment maturity, as shown in Table 6.

To illustrate the analytical utility of the three-dimensional framework, this study compares representative experiments from LAMP, RTAB-Map, and Swarm-SLAM [19,22,142]. The evaluation criteria focus on the scenarios and functionalities explicitly reported in the original publications, rather than the theoretical or latent capabilities of the software platforms. Scene complexity is defined by documented environmental challenges; autonomy levels are assessed based on task execution protocols and the extent of human intervention; and deployment maturity distinguishes between offline dataset replay and real-time operation in physical environments. Dimensions with inadequate reporting are marked as “insufficient evidence.”

LAMP conducted field trials across six underground mines, demonstrating mapping and deployment capabilities in constrained real-world environments. In specific instances, its loop-closure outlier rejection mechanism reduced end-to-end drift from 2.8 m to 0.2 m. However, challenges persist, including odometry degradation in featureless corridors, residual false loop closures, and bottlenecks associated with centralized communication architectures. The RTAB-Map case study involving the MIT Stata Center primarily employs data collected via teleoperated robots for replay-based evaluation, thereby facilitating assessments of localization-mapping accuracy and computational efficiency. Conversely, Swarm-SLAM achieved online collaborative mapping using three heterogeneous robots in a real-world parking garage, evidencing the feasibility of cooperative perception and inter-robot communication. Nevertheless, these results do not yet substantiate autonomous exploration or high-level task decision-making capabilities in complex subterranean environments.

These comparative cases reveal significant disparities in evidence regarding on-site simultaneous localization and mapping (SLAM) validation, collaborative map fusion, and higher-order autonomy. The three-dimensional framework effectively delineates environmental constraints, demonstrated functionalities, and deployment conditions, thereby identifying critical gaps in validation required for application in target scenarios. While this analysis underscores the framework’s explanatory value, its grading consistency and generalizability warrant further verification through expanded case studies and independent evaluations.

### 4.7. Comparison Technical Characteristics and Limitations of Representative Systems

Table 7 summarizes representative robotic mapping systems developed in recent years, along with their distinguishing technical characteristics. Collectively, advances in this field reflect three convergent evolutionary trends: (i) a transition from single-sensor paradigms to robust multi-sensor fusion; (ii) an expansion from purely geometric map representation toward semantically enriched mapping; and (iii) a shift from single-robot autonomy to coordinated multi-robot mapping [21,22,81]. Early work was largely anchored in established SLAM frameworks—most notably RTAB-Map—which prioritized reliable ego-motion estimation and consistent 3D geometric reconstruction in unstructured, unknown environments. In response to increasingly demanding real-world applications—such as dynamic urban settings, long-term operation, or perceptually degraded conditions—recent efforts have focused on next-generation systems including LAMP, LAMP 2.0, LOCUS, and EKF-LOAM [94,95,142,143]. These approaches integrate complementary sensor modalities (e.g., LiDAR, monocular/stereo vision, inertial measurement units, and wheel odometry) within principled estimation frameworks, thereby significantly improving localization robustness, mapping fidelity, and environmental adaptability.

Although state-of-the-art localization and mapping systems have achieved substantial improvements in positioning accuracy, real-time responsiveness, and environmental robustness, a systematic comparative analysis indicates that most current approaches remain predominantly oriented toward geometric reconstruction and metric map generation. Critical gaps persist in semantic scene understanding, context-aware autonomous decision-making, and dynamic risk assessment. Furthermore, operational deployment is hindered by persistent challenges—including communication reliability, cross-sensor temporal synchronization, and global consistency maintenance in large-scale or topologically complex environments. Crucially, much of the existing work is validated only under controlled laboratory conditions or narrowly defined field scenarios; there remains a marked scarcity of empirical evidence demonstrating long-term autonomy, reliable functionality in unstructured underground settings (e.g., tunnels, mines, or utility corridors), and scalability to commercial-grade applications. These limitations underscore the need for intensified research efforts targeting four interdependent technological pillars: deep multi-modal sensor fusion, semantically enriched mapping, resilient multi-robot coordination frameworks, and embodied intelligent reasoning—thereby enabling the evolution of confined-space robotic systems from high-fidelity geometric mapping toward fully integrated perception–decision–action loops.

### 4.8. Research Gap Matrix

The table above synthesizes the research focus, key technical limitations, and future development directions derived from the integration of all preceding analytical findings. It indicates that existing studies have established a relatively mature technical system centered on SLAM, LiDAR, single-robot systems, structured environments, and spatial mapping and localization, with notable advancements in map construction, information acquisition, and environmental perception. However, most of the research remains concentrated on the functions of spatial information acquisition and modeling, while exhibiting insufficient capabilities in intelligent decision-making processes such as semantic understanding of complex environments and autonomous decision-making. This reflects that although current research on exploration robots can address the question of “what the environment is,” it still faces challenges in comprehending “what the environment signifies” and tackling subsequent information processing issues. Table 8 summarizes the current research focus, existing limitations, and future research directions.

Further analysis reveals that future research directions are shifting from traditional geometric mapping toward intelligent environmental cognition, with the integration of cutting-edge AI tools into this research domain. Semantic Mapping represents a critical research direction for transcending conventional geometric mapping. Embodied AI and Autonomous Decision-making will further enhance robots’ capabilities in environmental understanding and autonomous decision-making. Multi-robot collaboration can improve exploration efficiency in unknown environments, while autonomous mapping in unstructured environments and Intelligent Risk Assessment will enhance robots’ safety and adaptability. In summary, future research on confined-space modeling robots will gradually evolve from a mapping-centric paradigm to the development of more intelligent autonomous systems.

## 5. Research Gaps and Future Directions

### 5.1. Research Gaps

Following an in-depth analysis of the statistical literature, it is evident that research on confined-space modeling robots has achieved substantial advancements in recent years, with a relatively well-established technical system in place. However, as emerging technologies continue to advance, and application scenarios become increasingly diversified, new research gaps have emerged. The identified research gaps, upon analysis, can be categorized into three dimensions: the perception gap, the intelligence gap, and the engineering deployment gap.

(1) Perception Gap

The perception layer constitutes the foundational basis for robotic spatial modeling and represents the most mature stage of current research. However, most studies remain confined to the acquisition of geometric information, with core objectives primarily focused on enhancing superficial performance metrics such as mapping accuracy and localization precision. In essence, robots are only capable of transmitting perceived raw data without engaging in further information processing. Additionally, existing research predominantly emphasizes obstacle detection and the characterization of unknown spatial topologies yet exhibits deficiencies in the identification of potential safety hazards and the analysis of traversable regions. This underscores the imperative of advancing from geometric mapping to semantic understanding [144,145,146,147].

(2) Intelligence Gap

With the advancement of robotic autonomous navigation technology, research focus is shifting toward intelligent development. However, a comprehensive analysis indicates that current modeling robots still rely on pre-set algorithms and manual processing, resulting in a relatively low level of intelligence. Most SLAM systems can only perform path planning based on existing algorithms, but struggle to dynamically adjust exploration strategies in real time according to actual spatial configurations or environmental changes and are even less capable of making autonomous decisions. Furthermore, current research is mostly applied in structured environments, such as industrial mines, tunnels, and indoor spaces, while there is a clear lack of research on unstructured environments like post-disaster ruins and geological spaces. The increasing demand for exploring such unknown scenarios provides impetus for the intelligent development of robots.

(3) Deployment Gap

From the perspective of experimental validation, current research predominantly relies on simulation and laboratory-based verification, while long-term deployment studies in real-world engineering environments remain conspicuously insufficient. Many algorithms, despite achieving high performance on simulation platforms, are still susceptible to uncertainties in practical application scenarios, often resulting in errors during task execution. Furthermore, existing research focuses primarily on the robot’s ability to complete mapping tasks, with insufficient attention paid to the robot’s intrinsic safety performance—such as autonomous return-to-home capability, operational duration planning, and communication constraints—factors that significantly impact exploration efficiency. Additionally, the absence of a unified data collection and testing platform, as well as standardized evaluation criteria, hinders cross-study comparisons and thus limits the comprehensive advancement of robotic systems. Therefore, enhancing the experimental deployment of robots in real-world environments constitutes a critical future research direction.

In summary, future research should prioritize addressing the gaps across the three dimensions analyzed above, advancing technical investigations into semantic perception, autonomous decision-making, risk identification, multi-robot collaboration, and real-scenario application deployment of robots—ultimately facilitating a technological transformation of modeling robots.

### 5.2. Future Research Directions

In conclusion, future research must transcend traditional mapping algorithms and integrate state-of-the-art AI functionalities to facilitate the convergence of autonomous robots with emerging technologies like digital twins, thereby supporting more reliable autonomous operation in the field of confined-space modeling robots.

(1) Semantic Mapping

In the domain of robotics, semantic mapping has emerged as the most prominent development trend for future semantic understanding and mapping technologies [148,149,150]. The application of semantic mapping in constrained environments remains hampered by low-texture surfaces, illumination variations, and dynamic interference. Furthermore, learning-based modules encounter significant challenges regarding cross-scenario generalization, geometric consistency, and long-term map maintenance. Consequently, future research must concurrently assess the reliability of both semantic recognition and simultaneous localization and mapping (SLAM), while explicitly representing task-relevant semantics and their associated uncertainties. It is critical to avoid conflating an increase in semantic label density with a genuine enhancement in environmental comprehension [151,152,153,154,155,156,157,158,159,160,161,162,163].

(2) Embodied AI

In recent years, Embodied AI has gradually emerged as a pivotal research direction within the field of robotics. A critical bottleneck in embodied artificial intelligence resides in ensuring robust integration across perception, planning, and control modules [164]. For navigation within narrow corridors and traversing irregular terrains, future validation protocols should explicitly incorporate physical constraints and environmental interaction dynamics [165]. Priority must be placed on assessing behavioral stability under conditions such as collisions, slippage, and perceptual anomalies, rather than relying exclusively on simulation-based task success rates to infer real-world applicability.

(3) Foundation Model

Against the backdrop of the rapid advancement of large language models, foundation models are progressively permeating and being applied in the field of robotics. Although foundation models facilitate semantic understanding and high-level planning, significant limitations persist regarding decision verifiability, computational overhead, and the assurance of safety constraints. In constrained environments, erroneous interpretations or infeasible action proposals can critically compromise task safety. Consequently, future research should prioritize architectures that synergize foundation models with geometric planning, safety monitoring mechanisms, and human-in-the-loop supervision. Rigorous validation is required to assess inference latency, anomaly handling protocols, and cross-scenario generalization capabilities [166,167].

(4) Multi-Robot Collaboration

Against the backdrop of an increasing number of future exploration missions in complex confined spaces, multi-robot collaboration is emerging as an important research direction [168,169,170,171]. Multi-robot collaboration is jointly constrained by communication and computational scalability. In underground or complex search-and-rescue environments, restricted communication further exacerbates the challenges of map fusion and maintaining state consistency. Future research should explicitly report metrics such as bandwidth limitations, communication interruptions, information exchange overhead, and recovery conditions, while critically evaluating collaborative gains under network-constrained scenarios. It is essential to avoid conflating decentralized architectures with guaranteed robustness in disconnected operations [172,173,174,175,176,177].

(5) Risk-aware Navigation

Given the high uncertainty of environmental risks in unknown spaces, traditional navigation algorithms have been primarily centered on optimal path planning or positioning accuracy. A significant disconnect persists between risk quantification and safety-critical decision-making. While SLAM-based local structural deformation monitoring yields early warnings in controlled, structured environments, such results do not substantiate the system’s capacity for collapse prediction or autonomous evacuation. Future research should integrate risk assessment with path feasibility, energy state management, and recovery strategies, rigorously evaluating false-positive rates, missed detections, decision latency, and actual mission completion outcomes [8,131].

(6) Autonomous Return

Autonomous return capability represents one of the current technical bottlenecks in robotics, while also serving as a critical safeguard for future engineering applications. In scenarios where robots encounter challenges such as insufficient power supply, communication disruption, or abrupt environmental changes, they need to autonomously plan a safe return trajectory to avoid loss. As such, autonomous return stands as an indispensable technological foundation for the future advancement of robotics [178].

(7) Digital Twin and Simulation

Digital twin technology enables the bidirectional mapping between simulated and real environments, serving as a reliable technical platform for robot algorithm development, task planning, and performance validation. Algorithmic performance is sensitive to sensor modalities, kinematic parameters, and environmental conditions; consequently, low trajectory error metrics on isolated datasets are insufficient to substantiate engineering reliability. Future benchmarking protocols should encompass tracking failures, recovery robustness, computational latency, and task safety, while rigorously distinguishing between offline dataset evaluations, controlled laboratory experiments, and real-world field deployments [166,179].

(8) Standardized Benchmark Platforms

Presently, within this research domain, in addition to addressing the core research challenges involved in the development of frontline robots, there exist notable bottlenecks in post-stage data collection. Specifically, the absence of unified data collection platforms and standardized evaluation systems has hindered the ability to conduct fair comparisons between different algorithms. While foundation models and embodied intelligence introduce novel paradigms for environmental understanding and task planning, the capabilities demonstrated in general robotics research do not inherently translate to reliable autonomy within confined spaces. Recent surveys emphasize that key unresolved challenges persist regarding cross-domain generalization, onboard computational constraints, errors at module interfaces, and the verifiability of learning-based decision-making systems [180,181,182,183,184,185,186,187,188].

In the future, robots will not only need to possess precise spatial modeling capabilities but also be equipped with environmental understanding, autonomous reasoning, independent decision-making, and long-term stable operation mechanisms—ultimately realizing the technological leap from perception-oriented mapping to intelligence-driven autonomous systems. This development trend not only delineates the future trajectory of robotics but also establishes a critical technical foundation for applications including underground mining, inspection of subterranean infrastructure, post-disaster rescue operations, and exploration of uncharted environments.

### 5.3. Limitations of the Study

First, the literature search was restricted to four academic databases, meaning the scope of coverage was constrained by each database’s indexing policies, search fields, and keyword configurations. Consequently, studies published in other venues or those utilizing divergent terminology to describe constrained environments may have been omitted. Thus, the findings presented herein reflect the sample included under a specific search strategy and should not be interpreted as an exhaustive representation of all research within this domain.

Second, this review did not systematically include preprint repositories, which may have resulted in the exclusion of cutting-edge findings that have not yet undergone formal peer review or publication. In rapidly evolving subfields such as foundational models, embodied intelligence, and multi-robot collaboration, this limitation could lead to an underestimation of both research activity and methodological diversity. As no systematic comparison was conducted with the excluded preprints, the magnitude of this selection bias cannot be quantified.

Third, bibliometric analyses are inherently influenced by metadata completeness, keyword normalization protocols, and parameter settings. Metrics such as citation counts and h-indexes are also sensitive to publication timing and citation accumulation windows; therefore, lower values for recent publications do not necessarily indicate inferior research quality or technical value. Furthermore, keyword co-occurrence and clustering primarily elucidate thematic associations among documents and cannot independently substantiate technical performance, causal relationships, or actual deployment readiness.

Finally, this study did not perform a unified assessment of the technical quality of the included works. Heterogeneity across studies regarding datasets, experimental scales, control methodologies, evaluation metrics, and reporting standards limits direct cross-study performance comparisons. The classifications of capability and deployment maturity presented here rely solely on evidence reported in the original texts and do not constitute independent verification of experimental reliability, reproducibility, or system safety. Accordingly, the conclusions should be understood as a synthesis of existing research characteristics and identified evidence gaps.

## 6. Conclusions

This study focuses on the current research status and future development trends of robotics for confined-space modeling. A systematic literature search was conducted across four authoritative academic databases—Web of Science, Scopus, IEEE Xplore, and ScienceDirect—resulting in the screening of 166 highly relevant publications. Leveraging bibliometric tools including VOSviewer, CiteSpace, and Bibliometrix, an in-depth and intuitive analysis of the field was performed, covering keyword clustering, technological evolution trajectory, historical development context, and international research landscape. Building upon the statistical analysis of basic publication metadata, multidimensional cross-cutting analyses were further carried out to evaluate the current technological maturity, identify existing application demands, and pinpoint future research gaps. This study thus provides a clear research framework and directional insights for the advancement of related fields.

The comprehensive analysis findings reveal that the field of confined-space modeling robotics has maintained a steady growth trajectory in recent years. SLAM (Simultaneous Localization and Mapping), LiDAR (Light Detection and Ranging), multi-sensor fusion, and autonomous navigation have gradually evolved into a mature technical system, serving as the foundational pillars of current research in this domain.

In terms of the overall research landscape, existing studies primarily prioritize the geometric mapping and localization capabilities of robots, while investigations into robotic intelligence remain relatively limited. Notably, research hotspots are undergoing a paradigm shift—from traditional information acquisition toward intelligent semantic understanding. Concurrently, the rapid advancement of artificial intelligence (AI) in recent years is increasingly permeating the robotics field; endowing robots with autonomous decision-making capabilities via AI has emerged as a core research objective for contemporary scholars.

Furthermore, cross-dimensional analyses across different countries globally demonstrate that research priorities vary significantly among nations, reflecting a strong correlation between a country’s research focus and its specific application demands.

Against the backdrop of the rapid advancement of emerging technologies including artificial intelligence (AI), digital twins, and embodied AI, research on confined-space modeling robotics is undergoing a notable shift toward semantic understanding. This transition empowers robots to adapt to a wider spectrum of application scenarios and enables their intelligent evolution toward autonomous decision-making, navigation, and judgment capabilities.

Moving forward, priority should be given to the integrated application of core technologies such as Semantic Mapping, Foundation Models, Embodied AI, Multi-Robot Collaboration, Risk-aware Navigation, and Autonomous Return to propel the technological transformation of exploratory robotics. Concurrently, the development of backend data platforms constitutes a prominent research gap and a critical future research objective. A specialized scenario-oriented data collection and analysis platform would facilitate the seamless integration of simulation studies and real-world application research, enabling cross-fertilization of research insights. This integration is expected to drive the comprehensive development of the field, mitigate application disparities across countries and regions, and enhance the feasibility of cross-regional collaboration.

The research gaps identified in this paper not only provide actionable research directions for subsequent scholars but also lay a theoretical foundation and offer technical references for the deployment of confined-space modeling robots in industrial, geological, and natural disaster response domains.

At present, confined-space modeling robots are in a critical phase of evolution, transitioning from basic map construction to intelligent exploration. With the deep integration of artificial intelligence (AI) into robotics technology in the future, robots will gradually acquire capabilities such as environmental understanding, autonomous decision-making, and long-term autonomous operation, ultimately emerging as a core technological enabler for the exploration of uncharted spaces. This trend not only delineates the future development trajectory of robotics but also is poised to further drive the intelligent advancement of domains including underground industrial operations, disaster rescue, and geological surveying.

## Figures and Tables

**Table 1:** Database-specific search strategies and retrieval details.

Database	Search Date	Publication Time Range	Search Fields and Complete Search Query	Records Retrieved
Scopus	17 April 2026	2016–2026	(“LiDAR SLAM” AND “confined environment”) AND (“topological mapping” OR “structure extraction”)	189
Web of Science	24 April 2026	2016–2026	(“visual recognition” OR “computer vision” OR “image classification”) AND (“systematic review” OR “systematic literature review”)	205
ScienceDirect	24 April 2026	2016–2026	(“computer vision” OR “visual recognition” OR “image recognition”)	197
IEEE Xplore	17 April 2026	2016–2026	(“LiDAR SLAM”) AND (“confined space”) AND (“robot”)	103

**Table 2:** Comparison of Major Bibliometric Analysis Tools.

Comparison Dimension	VOSviewer	CiteSpace	Bibliometrix
Core Strength	Network visualization	Trend detection & evolution	Comprehensive statistical analysis
Timeline Analysis	Limited	Excellent	Available
Burst Detection	Weak	Excellent	Limited
Data Processing Capability	Moderate	Moderate	Excellent
Suitability For Publication Figures	Excellent	Good	Good
Reproducibility	Moderate	Moderate	High

**Table 3:** Statistics of the Top 20 Most Cited Publications in the Field of 3D Modeling Robots for Confined Spaces.

Number	Title	Year	Cited By	Annual Citation
1	RTAB-Map as an open-source lidar and visual simultaneous localization and mapping library for large-scale and long-term online operation	2019	883	110.38
2	A Comparative Analysis of LiDAR SLAM-Based Indoor Navigation for Autonomous Vehicles	2022	283	56.6
3	M2DGR: A Multi-Sensor and Multi-Scenario SLAM Dataset for Ground Robots	2022	278	55.6
4	Complex urban dataset with multi-level sensors from highly diverse urban environments	2019	273	34.12
5	SegMap: Segment-based mapping and localization using data-driven descriptors	2020	195	27.86
6	SLOAM: Semantic Lidar Odometry and Mapping for Forest Inventory	2020	165	23.57
7	Robust LIDAR localization using multiresolution Gaussian mixture maps for autonomous driving	2017	162	16.2
8	Present and Future of SLAM in Extreme Environments: The DARPA SubT Challenge	2024	159	53
9	LAMP 2.0: A Robust Multi-Robot SLAM System for Operation in Challenging Large-Scale Underground Environments	2022	156	31.2
10	LAMP: Large-Scale Autonomous Mapping and Positioning for Exploration of Perceptually Degraded Subterranean Environments	2020	144	20.57
11	Unified Multi-Modal Landmark Tracking for Tightly Coupled Lidar-Visual-Inertial Odometry	2021	131	21.83
12	Swarm-SLAM: Sparse Decentralized Collaborative Simultaneous Localization and Mapping Framework for Multi-Robot Systems	2024	126	42
13	LOCUS: A Multi-Sensor Lidar-Centric Solution for High-Precision Odometry and 3D Mapping in Real-Time	2021	123	20.5
14	Ford Multi-AV Seasonal Dataset	2020	117	16.71
15	DiSCo-SLAM: Distributed Scan Context-Enabled Multi-Robot LiDAR SLAM with Two-Stage Global-Local Graph Optimization	2022	117	23.4
16	Stereo Visual Inertial LiDAR Simultaneous Localization and Mapping	2019	105	13.12
17	Building and optimization of 3D semantic map based on Lidar and camera fusion	2020	101	14.43
18	EKF-LOAM: An Adaptive Fusion of LiDAR SLAM With Wheel Odometry and Inertial Data for Confined Spaces with Few Geometric Features	2022	101	20.2
19	Multi-Sensor Fusion for Navigation and Mapping in Autonomous Vehicles: Accurate Localization in Urban Environments	2020	95	13.57
20	Robot-assisted mobile scanning for automated 3D reconstruction and point cloud semantic segmentation of building interiors	2023	94	23.5

**Table 4:** Statistics of the Top 20 Publications by Annual Average Citations in the Field of 3D Modeling Robots for Confined Spaces.

Number	Title	Year	Cited By	Annual Citation
1	RTAB-Map as an open-source lidar and visual simultaneous localization and mapping library for large-scale and long-term online operation	2019	883	110.38
2	A Comparative Analysis of LiDAR SLAM-Based Indoor Navigation for Autonomous Vehicles	2022	283	56.6
3	M2DGR: A Multi-Sensor and Multi-Scenario SLAM Dataset for Ground Robots	2022	278	55.6
4	Present and Future of SLAM in Extreme Environments: The DARPA SubT Challenge	2024	159	53
5	Swarm-SLAM: Sparse Decentralized Collaborative Simultaneous Localization and Mapping Framework for Multi-Robot Systems	2024	126	42
6	Complex urban dataset with multi-level sensors from highly diverse urban environments	2019	273	34.12
7	LAMP 2.0: A Robust Multi-Robot SLAM System for Operation in Challenging Large-Scale Underground Environments	2022	156	31.2
8	SegMap: Segment-based mapping and localization using data-driven descriptors	2020	195	27.86
9	Navigation system for orchard spraying robot based on 3D LiDAR SLAM with NDT_ICP point cloud registration	2024	77	25.67
10	SLOAM: Semantic Lidar Odometry and Mapping for Forest Inventory	2020	165	23.57
11	Robot-assisted mobile scanning for automated 3D reconstruction and point cloud semantic segmentation of building interiors	2023	94	23.5
12	DiSCo-SLAM: Distributed Scan Context-Enabled Multi-Robot LiDAR SLAM with Two-Stage Global-Local Graph Optimization	2022	117	23.4
13	Unified Multi-Modal Landmark Tracking for Tightly Coupled Lidar-Visual-Inertial Odometry	2021	131	21.83
14	LAMP: Large-Scale Autonomous Mapping and Positioning for Exploration of Perceptually-Degraded Subterranean Environments	2020	144	20.57
15	LOCUS: A Multi-Sensor Lidar-Centric Solution for High-Precision Odometry and 3D Mapping in Real-Time	2021	123	20.5
16	EKF-LOAM: An Adaptive Fusion of LiDAR SLAM With Wheel Odometry and Inertial Data for Confined Spaces with Few Geometric Features	2022	101	20.2
17	LOCUS 2.0: Robust and Computationally Efficient Lidar Odometry for Real-Time 3D Mapping	2022	88	17.6
18	VDBFusion: Flexible and Efficient TSDF Integration of Range Sensor Data	2022	85	17
19	Ford Multi-AV Seasonal Dataset	2020	117	16.71
20	Robust LIDAR localization using multiresolution Gaussian mixture maps for autonomous driving	2017	162	16.2

**Table 5:** Country-Level Technology Focus in Confined Space Modeling Robotics.

Country	Mapping	Perception	Intelligence
**China**	High	High	Low
**United States**	High	High	High
**Germany**	Medium	High	Medium
**Japan**	Medium	Medium	Low
**South Korea**	Medium	Medium	Low
**Canada**	Medium	Medium	Medium
**India**	Low	Low	Low
**Brazil**	Low	Low	Low

**Table 6:** Operational definitions and classification criteria for the three-axis analytical framework.

Analytical Dimensions	Level	Operational Definitions and Assessment Criteria
**Scene complexity**	C1: Lower	The experimental environment is static with favorable conditions and well-defined geometric features.
**Scene complexity**	C2: Medium	The literature explicitly identifies challenges such as geometric repetition, feature scarcity, low illumination, partial occlusion, or communication constraints; however, it does not substantiate significant environmental variability under the concurrent influence of multiple factors.
**Scene complexity**	C3: Higher	The literature explicitly documents the concurrent presence of multiple challenges, including dynamic obstacles, structural variations, complex terrain, or other significant uncertainties. The specific conditions substantiating this assessment must be enumerated individually.
**Autonomous capability**	A1: Mapping and Localization	The system performs pose estimation and mapping; however, motion is manually controlled or driven by an externally provided trajectory.
**Autonomous capability**	A2: Target Navigation	The system autonomously plans and executes motion towards externally specified goals.
**Autonomous capability**	A3: Self-directed exploration	The system autonomously selects exploration targets and updates the trajectory based on newly acquired environmental information.
**Autonomous capability**	A4: Task-level adaptation decision	The system autonomously adapts task objectives and determines control actions—such as continuation, suspension, or return—based on risk assessment, task status, and resource constraints.
**Deployment maturity**	M1: Simulation Verification	Algorithm and task validation is conducted exclusively within a simulated environment.
**Deployment maturity**	M2: Laboratory validation	Robot experimentation is conducted in artificially constructed, controlled physical environments.
**Deployment maturity**	M3: Semi-real environment validation	Evaluated in experimental facilities or simulated scenarios that exhibit realistic engineering characteristics, while environmental and task conditions remain under strict control.
**Deployment maturity**	M4: Field test	Field testing of robotic tasks in realistic operational scenarios.
**Deployment maturity**	M5: Commercial deployment	Demonstrated empirical evidence of real-world commercial deployment, as opposed to mere reports of application potential, productization plans, or isolated demonstrations.

Note: The above classifications are used for the literature case analysis in this article and do not represent a unified industry standard.

**Table 7:** Comparison of Representative Robotic Mapping Systems.

System	Year	Sensors	Application	Key Contribution	Main Limitation
RTAB-Map	2019	LiDAR + Camera	Large-scale mapping	Open-source SLAM framework	High computational cost
SegMap	2020	LiDAR	Localization	Segment-based place recognition	Limited generalization
SLOAM	2020	LiDAR	Forest mapping	Semantic LiDAR SLAM	Scenario-specific semantics
LAMP	2020	LiDAR + IMU	Underground	Large-scale subterranean SLAM	Communication dependence
LOCUS	2021	LiDAR + IMU	Real-time mapping	Robust LiDAR odometry	Limited semantic understanding
LAMP 2.0	2022	LiDAR + IMU	Underground	Multi-robot SLAM	High communication overhead
EKF-LOAM	2022	LiDAR + IMU + Wheel	Confined spaces	Adaptive sensor fusion	Drift in weak-feature environments
DiSCo-SLAM	2022	LiDAR	Multi-robot	Distributed graph optimization	Place-recognition ambiguity
Swarm-SLAM	2024	Multi-modal	Multi-robot	Decentralized collaborative SLAM	Synchronization challenges
DARPA SubT Review	2024	Multi-sensor	Extreme environments	Future direction of subterranean SLAM	Autonomy still limited

**Table 8:** Current Research Focus, Existing Limitations, and Future Directions.

Current Focus	Existing Limitation	Future Direction
**SLAM**	Only Geometry	Semantic Mapping
**LiDAR**	Perception Only	Embodied AI
**Single Robot**	Low Efficiency	Multi-Robot
**Structured Space**	Poor Generalization	Unstructured Space
**Mapping**	No Understanding	Autonomous Decision
**Localization**	No Risk Awareness	Intelligent Risk Assessment

## Data Availability

This review is based on published literature. The list of the 166 included studies is provided in Table S1 of the Supplementary Materials.

## Supplementary Materials

The following supporting information can be downloaded at https://www.mdpi.com/article/10.3390/s26217070/s1.
